# Population structure, serotype distribution and antibiotic resistance of *Streptococcus pneumoniae* causing invasive disease in Victoria, Australia

**DOI:** 10.1099/mgen.0.001070

**Published:** 2023-07-20

**Authors:** Charlie Higgs, Lamali Sadeesh Kumar, Kerrie Stevens, Janet Strachan, Norelle L. Sherry, Kristy Horan, Josh Zhang, Timothy P. Stinear, Benjamin P. Howden, Claire L. Gorrie

**Affiliations:** ^1^​ Department of Microbiology & Immunology, University of Melbourne, at the Peter Doherty Institute for Infection and Immunity, Melbourne, Victoria, Australia; ^2^​ Microbiological Diagnostic Unit Public Health Laboratory, Department of Microbiology & Immunology, University of Melbourne, at the Peter Doherty Institute for Infection and Immunity, Melbourne, Victoria, Australia; ^3^​ Department of Health, Victoria, Australia; ^4^​ Department of Infectious Diseases, Austin Health, Heidelberg, Victoria, Australia; ^5^​ Centre for Pathogen Genomics, University of Melbourne, Melbourne, Victoria, Australia

**Keywords:** *Streptococcus pneumonaie*, population genomics

## Abstract

*
Streptococcus pneumoniae* is a major human pathogen and can cause a range of conditions from asymptomatic colonization to invasive pneumococcal disease (IPD). The epidemiology and distribution of IPD-causing serotypes in Australia has undergone large changes following the introduction of the 7-valent pneumococcal conjugate vaccine (PCV) in 2005 and the 13-valent PCV in 2011. In this study, to provide a contemporary understanding of the IPD causing population in Victoria, Australia, we aimed to examine the population structure and prevalence of antimicrobial resistance using whole-genome sequencing and comprehensive antimicrobial susceptibility data of 1288 isolates collected between 2018 and 2022. We observed high diversity among the isolates with 52 serotypes, 203 sequence types (STs) and 70 Global Pneumococcal Sequencing Project Clusters (GPSCs) identified. Serotypes contained in the 13v-PCV represented 35.3 % (*n*=405) of isolates. Antimicrobial resistance (AMR) to at least one antibiotic was identified in 23.8 % (*n*=358) of isolates with penicillin resistance the most prevalent (20.3 %, *n*=261 using meningitis breakpoints and 5.1 % *n*=65 using oral breakpoints). Of the AMR isolates, 28 % (*n*=101) were multidrug resistant (MDR) (resistant to three or more drug classes). Vaccination status of cases was determined for a subset of isolates with 34 cases classified as vaccine failure events (fully vaccinated IPD cases of vaccine serotype). However, no phylogenetic association with failure events was observed. Within the highly diverse IPD population, we identified six high-risk sub-populations of public health concern characterized by high prevalence, high rates of AMR and MDR, or serotype inclusion in vaccines. High-risk serotypes included serotypes 3, 19F, 19A, 14, 11A, 15A and serofamily 23. In addition, we present our data validating seroBA for *in silico* serotyping to facilitate ISO-accreditation of this test in routine use in a public health reference laboratory and have made this data set available. This study provides insights into the population dynamics, highlights non-vaccine serotypes of concern that are highly resistant, and provides a genomic framework for the ongoing surveillance of IPD in Australia which can inform next-generation IPD prevention strategies.

## Data Summary

Genome sequences are deposited in GenBank under BioProject PRJNA857543 and the accession numbers and sample data is available in the Supplementary Material (Data S1). All supporting data, code and protocols have been provided within the article or through supplementary data files.

**Impact Statement**

This study provides a comprehensive characterization of invasive pneumococcal disease isolates collected in Australia between 2018 and 2022 based on a combination of genomic and phenotypic data. Despite having a national immunization programme using the 13-valent pneumococcal conjugate vaccine (13v-PCV), vaccine serotypes represented 35 % of isolates. Within the highly diverse collection, the study identified six high-risk sub-populations of public health concern based on characteristics, such as high prevalence, high rates of AMR and MDR and serotype inclusion in vaccines. These high-risk serotypes included serotypes 3, 19F, 19A, 14, 11A, 15A and serofamily 23. We present our data validating seroBA for *in silico* serotyping to facilitate ISO-accreditation of this test in routine use in a public health reference laboratory and have made this data set available. Here we present a genomic framework for the ongoing surveillance and contribute to the understanding of IPD epidemiology in Australia within a global context.

## Introduction

*Streptococcus pneumoniae* is a Gram-positive opportunistic pathogen that colonizes the mucosal surface of the upper respiratory tract of humans [1]. *S. pneumoniae* can cause a range of conditions from asymptomatic colonization to invasive pneumococcal disease (IPD), the latter defined as the identification of pneumococci in a normally sterile specimen, such as blood or cerebrospinal fluid (CSF), including bacteraemia and meningitis. *S. pneumoniae* is a leading cause of pneumonia worldwide and the World Health Organisation estimates that globally, more than 300 000 children under 5 years of age die from *S. pneumoniae* infections every year [2, 3]. The rate of IPD in a given population is heavily dependent on the age group, with young children and elderly people having the highest incidence [4, 5].

The virulence of *S. pneumoniae* is highly dependent on the polysaccharide capsule, encoded by the capsular polysaccharide (*cps*) locus, with some capsule types being more associated with virulence than others [1]. Currently, over 100 capsule types or serotypes have been identified. However, due to the introduction of pneumococcal vaccines, which target common invasive serotypes, the epidemiology and distribution of *S. pneumoniae* serotypes have undergone large changes over the last two decades [6]. The first pneumococcal vaccine included as part of the routine childhood vaccination schedule in Australia was the 7-valent pneumococcal conjugate vaccine (7vPCV) in 2005 and targeted serotypes 4, 6B, 9V, 14, 18C, 19F and 23F. Although the 7vPCV resulted in a reduction in IPD incidence, cases caused by serotypes not included in the vaccine began to increase following its introduction, a process known as serotype replacement [7]. In 2011, the 7vPCV was changed to a 13 valent vaccine (13v-PCV) which, in addition to the serotypes included in the 7vPCV, targeted 1, 3, 5, 6A, 7F and 19A. In Australia, it is recommended that all children receive a three-dose schedule of the 13PCV at 2, 4 and 12 months of age and that all persons receive an additional single dose at 70 years of age [8]. In addition, there is a 23-valent pneumococcal polysaccharide (23vPPV) vaccine that is recommend for those with medical risk factors and the elderly [8]. The 23vPPV includes all serotypes included in the 13cPCV as well as serotype 2, 8, 9 N, 10A, 11A, 12F, 15B, 17F, 20B, 22F and 33F.

The introduction of these vaccines resulted in a decrease in IPD case rates but in recent years these numbers have been approaching pre-vaccine levels [9]. Of particular concern are the large numbers of IPD cases with serotypes contained in the PCVs, especially those found in the 13v-PCV but not in the 7v-PCV [9]. These cases are known as pneumococcal conjugate vaccine failures and can occur for a variety of reasons, including the poor immunogenicity of the serotype antigen and the heterogeneity of the *cps* locus, which can reduce the protection provided by the antigens contained in the PCVs [10–12]. *S. pneumoniae* is also known to undergo serotype switching, whereby the *cps* locus is altered through recombination, resulting in an alternate serotype that can enable vaccine evasion [13, 14].

In addition to the challenges raised by vaccine failures, antibiotic resistance in *S. pneumoniae* is also a growing concern. Once universally susceptible to penicillin, the first penicillin-resistant *S. pneumoniae* was reported in Australia in 1967, with beta-lactam resistance steadily increasing since then, in parallel with increasing penicillin-resistance globally [15, 16]. Increasingly, resistance to macrolides and tetracyclines have also been noted globally [17–19].

The ability of *S. pneumoniae* to readily recombine and exchange genetic material, particularly in relation to genes involved in determining serotype and antibiotic resistance, necessitates the use of whole-genome sequencing (WGS) as lineages can rapidly adapt and change their phenotypic characteristics. This means that typing methods like serotype do not accurately predict population structure. Although well-established genomic categorization methods such as multi-locus sequence type (MLST) provide some indication of the genomic background of an isolate, newer typing schemes that take into account more of the bacterial genome and provide more international context, such as Global Pneumococcal Sequence Cluster, are increasingly being used [20]. In addition, WGS allows for the identification of recombination and can be used to predict serotype, with *in silico* typing tools such as seroBA [21], and to detect the presence of known antimicrobial resistance genes and mutations.

In this study, we applied genome-scale analysis and comprehensive antimicrobial susceptibility testing to examine the population structure, prevalence of antimicrobial resistance and prevalence of vaccine failure events in the IPD causing isolates collected in Victoria, Australia between 2018 and 2022. In addition, our study aims to define a genomics framework that can be used as the basis for future public health surveillance and inform next-generation IPD prevention strategies. This includes validation of the *in silico* serotyping tool seroBA [21] for routine use in the public health microbiology setting.

## Methods

### Study design and data set

**Fig. 1.**
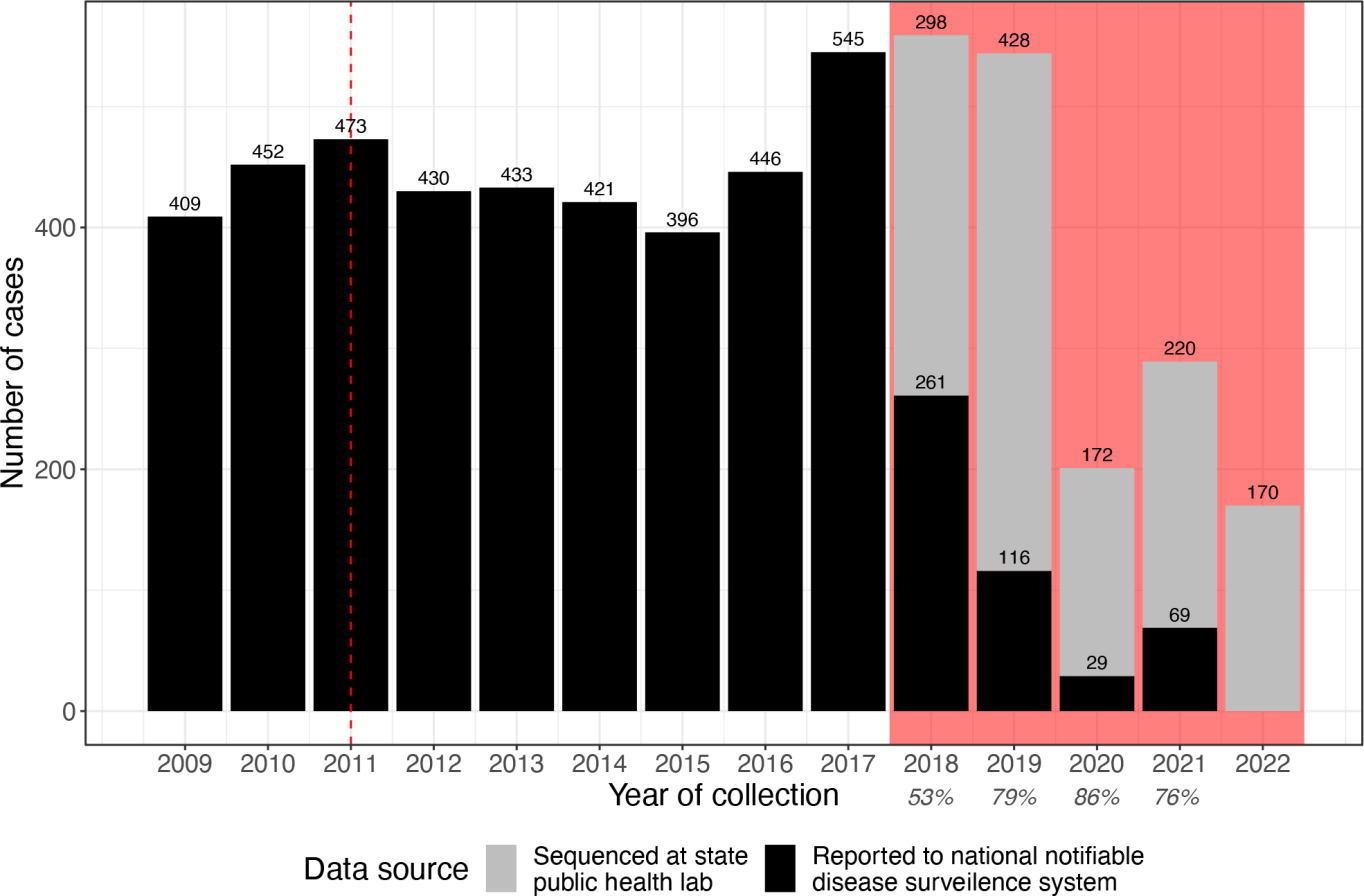
Count of invasive pneumococcal disease (IPD) cases over time. Case counts are based on IPD cases reported to the National Notifiable Disease Surveillance System (NNDSS) by the Victorian Department of Health and the number of *Streptococcus pneumoniae* isolates sequenced at the state public health included as part of this study (subset of the NNDSS number). The percentage of NNDSS cases represented by study isolates is listed below the study years. Note that for NNDSS data the state totals for Victoria and Tasmania are grouped together and the notification data is not available for 2022. Shaded red area indicates the study period (July 2018 to June 2022). Dashed red line indicated when the Australian vaccine schedule was updated to include the 13-valent pneumococcal conjugate vaccine in 2011 (previously the 7-valent vaccine was used).

Pneumococcal isolates included in this study were collected from all cases of invasive pneumococcal disease (IPD) between August 2018 and June 2022 (inclusive) as part of the state laboratory-based surveillance in Victoria, Australia (population 6.5 million in 2021 [22]). An IPD case was defined as pneumococci isolated from a normally sterile site [23] and notification is mandatory for diagnostic laboratories and diagnosing clinicians. The number of IPD notifications made to the National Notifiable Disease Surveillance System (NNDSS) by the Victorian Department of Health within the same period was 559 cases in 2018, 544 cases in 2019, 201 cases in 2020, and 289 cases in 2021 (Figs 1 and S1, available in the online version of this article). Following exclusion of duplicate isolates from the same patient and isolates with non-viable cultures, missing data and PCR-positive/culture-negative cases, a total of 1288 IPD isolates were included in this study; 298 from 2018 (53 % NNDSS cases), 428 from 2019 (79 % NNDSS cases), 172 from 2020 (86 % NNDSS cases), 220 from 2021 (76 % NNDSS cases) and 170 from 2022. Note that the study period was only for the second half of 2018 while NNDSS data spans the whole year and there was no NNDSS data currently available for 2022. Most isolates were isolated from blood (95 %) with the remaining isolates from cerebrospinal fluid (CSF) (1 %) or other (4 %). A list of samples included in the study and their associated metadata is available in Data S1 (available with the online version of this article) and a list of NNDSS cases is available in Data S2.

Vaccine status of patients was obtained through the Victorian Department of Health, and was only available for those <5 years old or >50 years old. The number of doses and type of vaccine was recorded. Patients were classified as ‘Unknown’ if their vaccination status was not known, ‘Not vaccinated’ if they had no doses of any pneumococcal vaccine, ‘Serotype not covered’ if they had received any number of doses of a *S. pneumoniae* vaccine that did not include coverage against the serotype of their infection, ‘Primary dose’ or ‘Secondary dose’ if they had received one or two doses of the 13v-PCV or 23v-PPV, respectively, and the serotype of their infection was covered by their respective vaccine or ‘Fully vaccinated’ if they had received three or more doses of a vaccine that provided coverage against the serotype of their infection.

### Antimicrobial susceptibility testing

Antimicrobial susceptibility testing was performed using Sensititre broth microdilution (BMD) using STP6F plates (Thermo Fisher Scientific, Waltham, MA, USA). Bacterial isolates were cultured overnight on horse blood agar (HBA) (incubated at 35–37⁰C in CO_2_), then 100 ul of a 0.5 McFarland suspension of organism in Mueller–Hinton broth was added to cation-adjusted Mueller–Hinton broth with 4 % lysed blood. 100 ul of this suspension was used to inoculate the STP6F plate, which was incubated for 24 h at 35–37⁰C in CO_2_. The MIC was recorded using the fluorescence based OptiRead system (Thermo Fisher Scientific, Waltham, MA, USA). The MIC results were interpreted and categorized according to Clinical and Laboratory Standards Institute (CLSI) breakpoints 2022 [24]. The following antimicrobials were included in the STP6F panel: amoxicillin/clavulanic acid, azithromycin, cefepime, cefotaxime, ceftriaxone, cefuroxime, chloramphenicol, clindamycin, ertapenem, erythromycin, levofloxacin, linezolid, meropenem, moxifloxacin, penicillin, tetracycline, trimethoprim/sulfamethoxazole, and vancomycin.

### Whole-genome sequencing

Genomic DNA was extracted from bacterial isolates using a JANUS automated workstation (PerkinElmer) and Chemagic magnetic bead technology (PerkinElmer). Genomic DNA libraries were then prepared using the Nextera XT kit according to the manufacturer’s instructions (Illumina). Whole-genome sequencing was performed on the Illumina NextSeq platform using 2×150 bp paired end chemistry.

### Genome quality control, assembly and mapping

The Bohra (v.2.0.0) (https://github.com/kristyhoran/bohra) pipeline was used to conduct quality control and to assemble the isolate genomes. Reads underwent quality control (default thresholds), genomes were assembled using skesa [25] (v. 2.4.0) within the shovill (https://github.com/tseemann/shovill) wrapper (v1.0.4) using default parameters. All core-genome alignments were performed using snippy (v4.4.5, https://github.com/tseemann/snippy) with default parameters (minfrac=0 and mincov=10). The reference genome used for all analyses was ASM966447v1 (GCA_009664475.1) as it is an Australian isolate collected locally (collected 2014, serotype 19A, ST199).

### Typing

Serotyping was performed *in silico* using seroBA (v. 1.0.2) [21] for all isolates. A subset of isolates collected between 2003 and 2019 (*n*=765) were used for validation of seroBA and were serotyped phenotypically using the Quellung Reaction [26] and *in silico* using seroBA. The Quellung was performed as previously described [26]. A full list of the isolates used for the seroBA validation is provided in Data S3. Multi-locus sequence typing (MLST) was performed using mlst (v. 2.19.0) (https://github.com/tseemann/mlst) and the pubMLST database (https://pubmlst.org/) [27] for *S. pneumoniae *. A Global Pneumococcal Sequence Cluster (GPSC) was assigned for all isolates using the *k*-mer based clustering method, PopPUNK (v2.4) [28], and the Global Pneumococcal Sequencing (GPS) database (GPSC_v6) (https://www.pneumogen.net/gps/training_command_line.html) [20].

### Genetic determinants of antimicrobial resistance

AbritAMR v1.0.7 [29] was used to screen each genome assembly for genetic determinants of resistance. This tool is a wrapper for the NCBI AMRFinderPlus tool [30] that has outputs adapted for clinical and public health microbiology reporting. Results were expressed in terms of major and minor errors. Major error=number (%) of susceptible isolates in which a resistance determinant was identified; very major error=number (%) of resistant isolates in which a resistance determinant was not identified (i.e. unexplained resistance).

### Phylogenetic trees

All phylogenetic trees were inferred using IQtree [31] (v. 2.1.4) with constant sites, 1000 bootstraps and a generalized time-reversible model of evolution (GTR +G4). The reference used for all whole-genome alignment phylogenies was ASM966447v1 (https://www.ncbi.nlm.nih.gov/assembly/GCA_009664475) (ST199, serotype 19A and 2 090 792 bp). For the species alignment, the core alignment length was 1 259 811 and the core SNP alignment was 102 339 sites. Recombination masking was not performed on whole-genome alignments due to the small size of the resulting alignment. All trees were midpoint rooted and visualized in R (v. 4.1.2, https://www.R-project.org/) using a combination of phangorn [32], ggtree [33] and ggnewscale (https://eliocamp.github.io/ggnewscale/).

### Data visualization and statistics

Figures were generated in R (v. 4.1.2) (https://www.R-project.org/) using the tidyverse suite [34] and UpSetR [35].

## Results

### The Victorian IPD causing population is highly diverse

Despite the introduction of the 13v-PCV in 2011, the number of IPD cases in Victoria remained approximately 400 per year from 2011 to 2016 before increasing to approximately 550 cases per year between 2017–2019. There was a marked decrease in cases during the COVID pandemic (including several prolonged lockdowns in Victoria) during 2020 and 2021, however the number of cases for the first half of 2022 suggests that case counts are returning to pre-pandemic levels. For all full calendar years of the study period (2019–2021), the number of cases reported to the NNDSS compared to the number of cases, which had sequence data available ranged from 76 –86 % (Fig. 1).

**Fig. 2.**
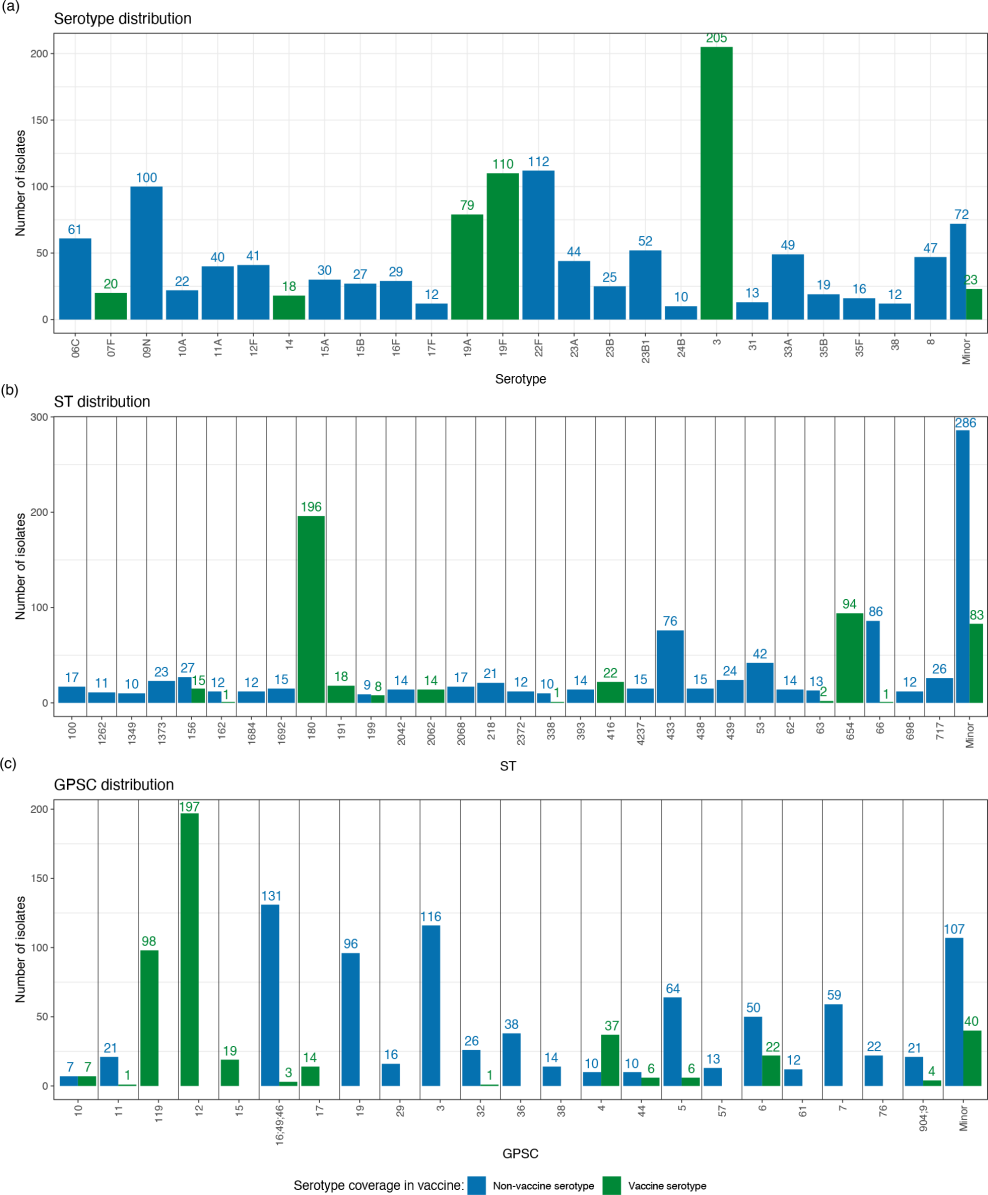
Counts of isolates over the study period based on (a) serotype, (b) sequence typing (ST) and (c) global pneumococcal sequence cluster (GPSC). Counts are coloured based on if the serotype of the isolate is covered by the 13-valent pneumococcal conjugate vaccine, blue for non-vaccine serotype and green for vaccine serotype.

Among the 1288 *S *. *pneumoniae* isolates included in the study, 52 different serotypes were identified. Serotypes included in the 13v-PCV represented 35.3 % (*n*=455) of isolates; the most common serotypes were 3 (15.9 %, *n*=205), 19F (8.5 %, *n*=110) and 19A (6.1 %, *n*=79). Serotypes included in the 23v-PPV but not in the 13v-PCV (add-23v-PPV) represented 31.4 % (*n*=405) of isolates; the most common were 22F (8.7 %, *n*=112), 9 N (7.8 %, *n*=100) and 8 (3.6 %, *n*=47). Serotypes not included in either vaccine represented 33.2 % (*n*=428) of isolates; the most common were 33A (3.8 %, *n*=49), 6C (4.7 %, *n*=61) and subtype 23B1 (4.0 %, *n*=52). There were 25 isolates with multiple possible serotypes due to a ‘tie’ in scores, as identified by seroBA [21] (Fig. 2a and Data S1).

There was high concordance between the phenotypic serotyping method (Quellung reaction) and the *in silico* method seroBA with 96.6 %(739 of 765 isolates) recording the same serotype. Of the remaining 26 discordant serotype results, there were 19 serotype combinations with no combination greater than four in frequency (Table S1 and Data S3). We do note that 33 isolates were Quellung serotype 23B/SeroBA subtype 23B1 and Quellung serotype 6B/SeroBA subtype 6E. The subtypes 23B1 and 6E can only be identified using *in silico* typing as they are not serologically distinct from 23B or 6B, respectively.

The 1288 isolates included 203 distinct sequence types (STs), the most common of which were ST180 (15.2 %, *n*=196), ST654 (7.3 %, *n*=94), ST66 (6.8 %, *n*=87) and ST433 (5.9 %, *n*=76) (Fig. 2b). The majority of STs (87 %, *n*=176) were associated with one serotype, with the maximum number of serotypes within a single ST being five (ST156 and ST160). The diversity of STs within serotypes was much greater. There were eight serotypes (15 %) that contained only one ST, three serotypes contained more than 10 serotypes (serotype 19A, 6C and 15B) and the largest number of STs in one serotype was 18 (serotype 19A and 6C) (Fig. 2).

Using the Global Pneumococcal Sequencing Project Cluster (GPSC) scheme to classify isolates, 70 GPSCs were identified, two of which were multiple predefined GPSCs joined together (GPSC 16;49;46 and 904;9). The most common GPSCs identified were 12 (15.3 %, *n*=197), 16;49;46 (10.4 %, *n*=134) and 3 (9.0 %, *n*=116) (Fig. 2c). The majority of GPSCs contained one serotype (*n*=47, 67 %) and one ST (*n*=40, 57 %) with serotype diversity ranging from 1 to 13 and ST diversity ranging from 1 to 18 (Fig. S2).

**Fig. 3.**
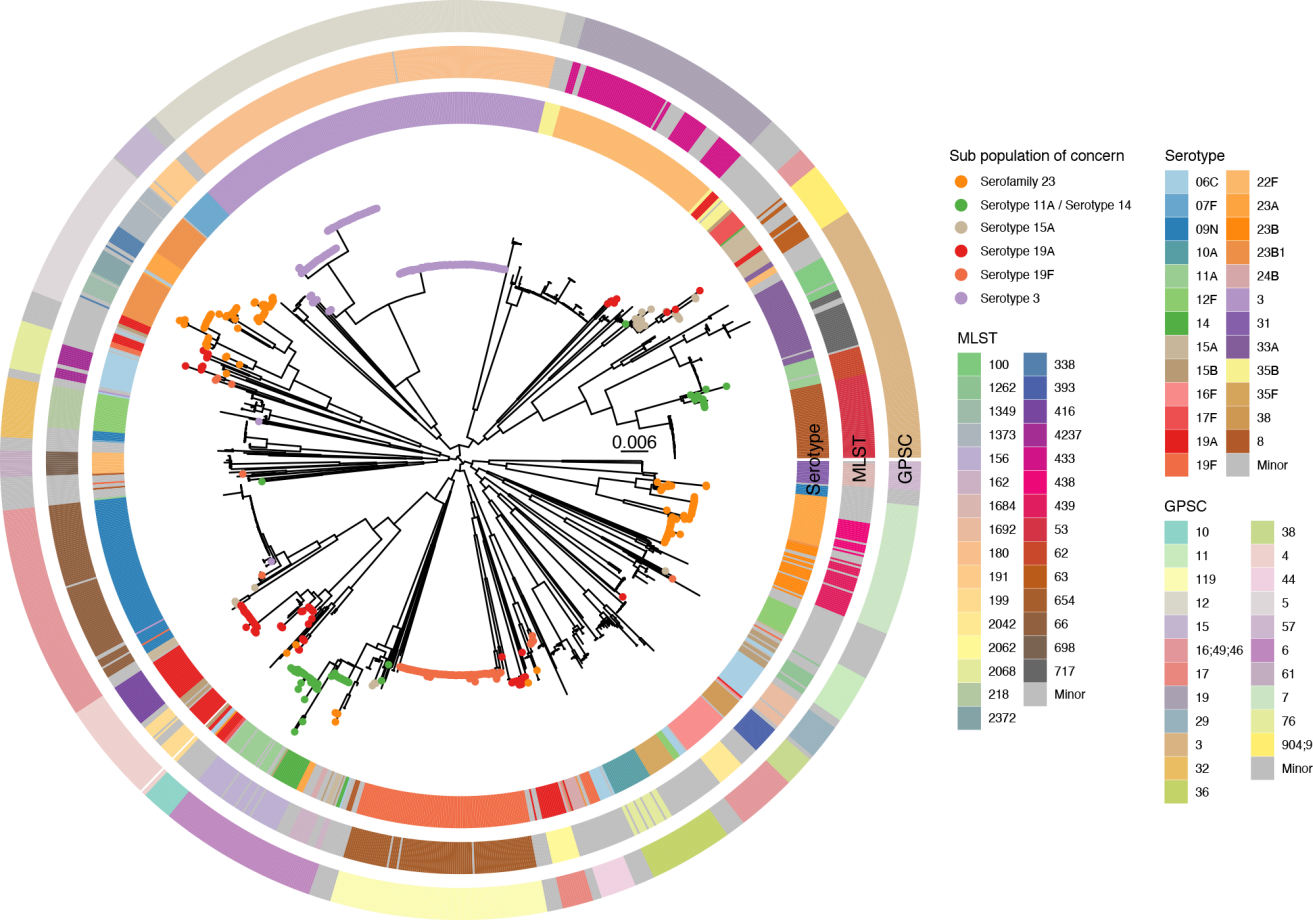
Midpoint-rooted maximum-likelihood phylogenetic tree of all study isolates (*n*=1,288). Tree tips are coloured if they are contained within any of the sub-populations of concern. Note that serotype 11A/14 also includes all other isolates of ST156. Minor serotypes, STs and global pneumococcal sequence cluster (GPSC) were defined as those that contained less than ten isolates over the study period. The reference was ASM966447v1 (GCA_009664475.1); collected 2014, serotype 19A, ST199.

A core-genome phylogenetic tree of all study isolates further illustrates the highly diverse nature of the population (Fig. 3).

### Antimicrobial resistance

Susceptibility testing using the broth microdilution method identified 23.8 % (*n*=308) as resistant to at least one antibiotic. Penicillin resistance, when using meningitis breakpoints (*S*≤0.06 µg ml^−1^, *R*≥0.12 µg ml^−1^), was identified in 261 (20.3 %) isolates, but, as expected, was much less prevalent when using the non-meningitis (*S*≤2 µg ml^−1^, *I*=4 µg ml^−1^, *R*>4 µg ml^−1^) and oral (*S*≤0.06 µg ml^−1^, 0.12 µg ml^−1^ ≤*I*≥1 µg ml^−1^, *R*≥2 µg ml^−1^) breakpoints due to the higher MIC breakpoints for resistance [4(0.3 %) and 65(5 %) isolates, respectively]. Of the 14 cerebrospinal fluid samples in the data set, 6(43 %) were resistant to penicillin. Resistance to third-generation cephalosporin antibiotics (cefotaxime and ceftriaxone) using meningitis breakpoints was no greater than 3 % of all isolates and resistance to cefuroxime was 6 % (*n*=77). Resistance to macrolide antibiotics was 11.3 % (*n*=145) for erythromycin, 10.8 % (*n*=123) for azithromycin and 8.2 % (*n*=106) for clindamycin. All isolates were susceptible to ertapenem, vancomycin and linezolid (Table 1).

**Table 1.** Antimicrobial susceptibility results of 1286 *S *. *pneumoniae* isolates. Amox-clav acid refers to amoxicillin and clavulanic acid and trimeth-sulpha refers to trimethoprim-sulfamethoxazole. NAs indicate that there is no such breakpoint for that antibiotic. Breakpoints are based on the 2022 CLSI guidelines. Men refers to meningitis breakpoints, non-men to non-meningitis breakpoints and oral to orla breakpoints

Antibiotic subclass	Antibiotic (men/non-men breakpoint used, if applicable)	Total no. isolates tested	No. sensitive isolates (%)	No. intermediate isolates (%)	No. resistant isolates (%)
Beta-lactam	Amox-clav acid	1176	1145 (97.4 %)	17 (1.4 %)	14 (1.2 %)
	Cefepime (men)	1286	1205 (93.7 %)	41 (3.2 %)	40 (3.1 %)
	Cefepime (non-men)	1286	1246 (96.9 %)	36 (2.8 %)	4 (0.3 %)
	Cefotaxime (men)	1286	1218 (94.7 %)	44 (3.4 %)	24 (1.9 %)
	Cefotaxime (non-men)	1286	1262 (98.1 %)	24 (1.9 %)	0 (0 %)
	Ceftriaxone (men)	1285	1200 (93.4 %)	46 (3.6 %)	39 (3.0 %)
	Ceftriaxone (non-men)	1285	1246 (97.0 %)	35 (2.7 %)	4 (0.3 %)
	Cefuroxime	1286	1190 (92.5 %)	19 (1.5 %)	77 (6.0 %)
	Ertapenem	1286	1278 (99.4 %)	8 (0.6 %)	0 (0 %)
	Meropenem	1286	1220 (94.9 %)	44 (3.4 %)	22 (1.7 %)
	Penicillin (men)	1285	1024 (79.7 %)	na	261 (20.3 %)
	Penicillin (non-men)	1285	1251 (97.4 %)	30 (2.3 %)	4 (0.3 %)
	Penicillin (oral)	1285	1024 (79.8 %)	196 (15.2 %)	65 (5.1 %)
Chloramphenicol	Chloramphenicol	1286	1265 (98.4 %)	na	21 (1.6 %)
Glycopeptide	Vancomycin	1286	1286(100 %)	na	na
Macrolide	Azithromycin	1135	1012 (89.2 %)	0 (0 %)	123 (10.8 %)
	Clindamycin	1286	1172 (91.1 %)	8 (0.6 %)	106 (8.2 %)
	Erythromycin	1286	1133 (88.1 %)	8 (0.6 %)	145 (11.3 %)
Oxazolidinone	Linezolid	1286	1118 (100 %)	na	na
Quinolone	Levofloxacin	1285	1281 (99.7 %)	2 (0.2 %)	2 (0.2 %)
	Moxifloxacin	1285	1280 (99.6 %)	4 (0.3 %)	1 (0.1 %)
Tetracycline	Tetracycline	1286	1129 (87.8 %)	22 (1.7 %)	135 (10.5 %)
Trimethoprim	Trimeth-sulpha	1286	1003 (78.0 %)	122 (9.5 %)	161 (12.5 %)

Among common serotypes (serotypes with more than ten isolates total over the study period), resistance to any antibiotic (using meningitis breakpoints where available) was most common in serotypes 14 (100 %, *n*=18), 15A (80 %, *n*=24), 11A (65 %, *n*=28), 33A (59 %, *n*=29) and subtype 23B1 (96 %, *n*=51) (Table S2). Serotype 14 is included in the 13v-PCV and 23v-PPV and 11A is included in the 23v-PPV, whilst the remaining serotypes are not included in either vaccine. Among common STs (STs with more than ten isolates total over the study period), there were six STs that had 100 % resistance to penicillin using meningitis breakpoints: STs 1349 (*n*=10), 1373 (*n*=23), 156 (*n*=42), 2062 (*n*=14), 2372 (*n*=12) and 338 (*n*=11) (Table S3).

**Fig. 4.**
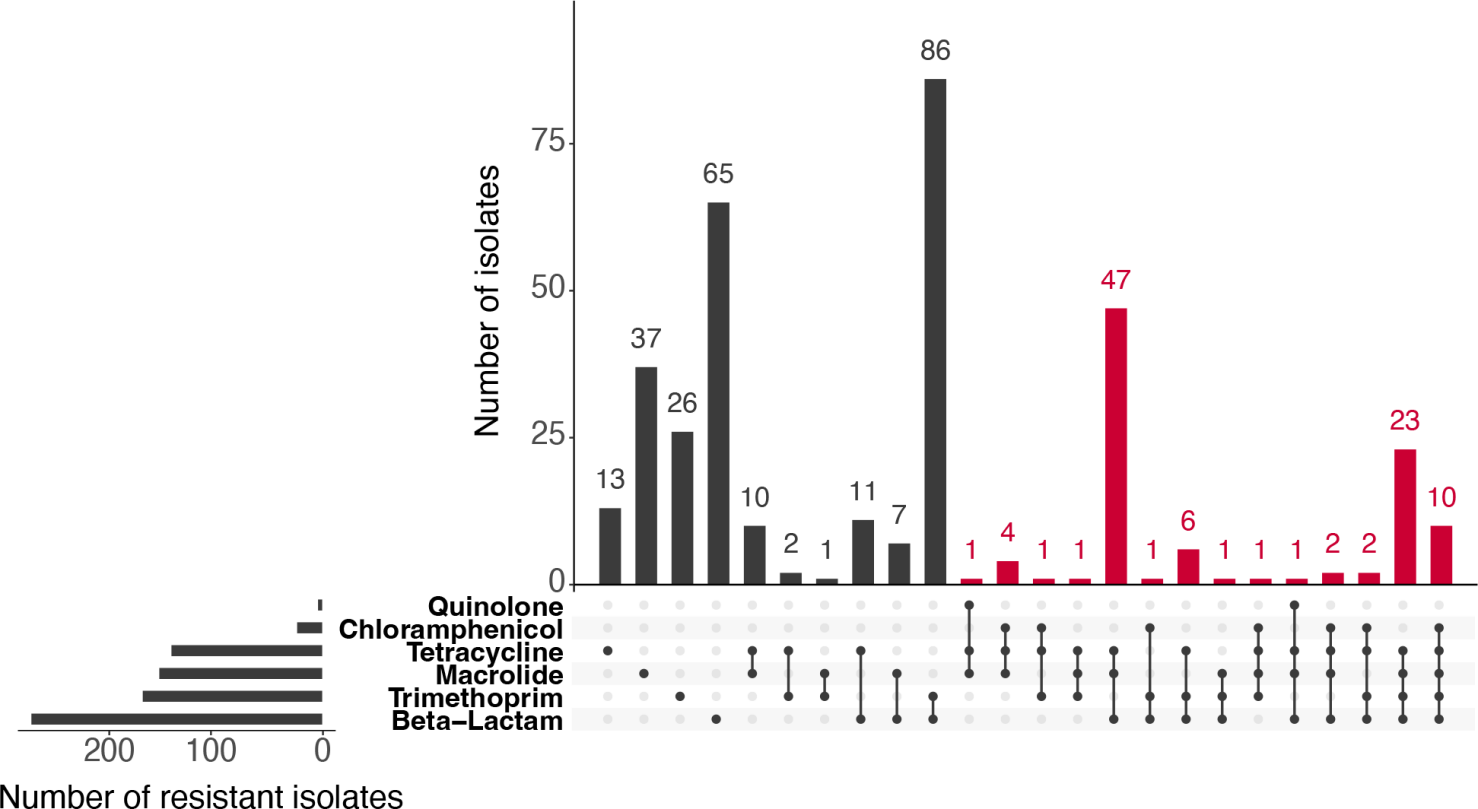
Upset plot of the distribution of multidrug-resistant isolates. Isolates have been grouped by class of antibiotic. Multidrug resistant (MDR) are defined as being resistant to three of more classes of antimicrobials and are highlighted in the plot in red.

Multidrug resistance (MDR) (resistant to three or more antibiotic classes) was identified in 7.8 % (*n*=101) of all isolates. The most common combinations of antibiotic resistance was resistance to beta-lactams, macrolides and tetracyclines (69 % of MDR isolates, *n*=70), with 23 of these additionally resistant to trimethoprim/sulfamethoxazole (Fig. 4). The serotypes with the largest numbers of MDR isolates, when using the resistant breakpoints only, were 15A (*n*=24, 100 % of resistant isolates MDR), 19A (*n*=21, 51.2 % of resistant isolates MDR) and 23A (*n*=14, 87.5 % of resistant isolates MDR). Despite having a large proportion of isolates resistant, isolates from serotype 14 (17 % of resistant isolates MDR, *n*=3), 11A (11 % of resistant isolates MDR, *n*=3) and subtype 23B1 (0 % of resistant isolates MDR, *n*=0) were not likely to be MDR (Table S2).

There was poor correlation between AMR genotype and phenotype with many antibiotics having high proportions of major and very major errors (Table S4). The beta-lactam antibiotics had consistently high (>20 %) major error rates.

### Vaccine failures

To investigate potential vaccine failure events, the vaccination status of IPD cases from June 2018 to December 2021 was assessed. Of the 1118 cases, vaccination status was known for 742 cases (66.4 %) and 462 (62.3 %) of these had received at least one dose of a vaccine. The cases that had received at least one dose were further classified as those that received at least one dose of a vaccine that targeted the serotype of their infection (*n*=223, 48.3 %) and of these cases 34 (15.2 %) had received a full three doses of the vaccine so were classified as fully vaccinated (Table 2).

**Table 2.** Breakdown of vaccination status and infection serotype for cases. Note that vaccination status was only requested for a subset of isolates (June 2018 and December 2021, *n*=1118) due to data availability

Total cases (*n*=1118)	*N*	%
Vaccination status NOT known:	376	33.6
Vaccination status known:	742	66.4
Received NO IPD vaccines:	280	37.7
Received any IPD vaccines:	462	62.3
Case’s serotype NOT covered by vaccine:	239	51.7
Case’s serotype covered by vaccine:	223	48.3
Primary dose (13 v):	39	17.5
Primary dose (23 v):	126	56.5
Secondary dose (13 v):	4	1.8
Secondary dose (23 v):	20	9.0
Fully vaccinated (three doses):	34	15.2
Serotype 14 (13v)	2	5.9
Serotype 19A (13 v)	6	17.6
Serotype 19F (13 v)	13	38.2
Serotype 22F (23 v)	1	2.9
Serotype 23F (13 v)	1	2.9
Serotype 3(13v)	11	32.4

These 34 cases were classified as vaccine failure events and account for 7.4 % of cases that had received any vaccine doses. The serotypes with the largest number of vaccine-failure events were serotype 19F (*n*=13), 3 (*n*=11) and 19A (*n*=6). These are also the most numerous serotypes in the study population that are included in the 13v-PCV vaccine (Table 2). To assess which serotypes were most likely to evade the vaccine, we determined the proportion of total cases of each serotype that were vaccine-failure events. Serotype 23F had the highest proportion of vaccine-failure cases (50 %) but only had two cases over the study period. The serotypes with the next highest proportion of vaccine failure cases were 14 and 19F (11.1 and 11.8 % cases of the respective serotypes) (Table 3). Most vaccine-failure events occurred in people below the age of 12 (Fig. S3).

**Table 3.** Breakdown of vaccination failure events. Note that vaccination status was only requested for a subset of isolates (June 2018 and December 2021, *n*=1,118) due to data availability

Serotype (vaccine)	Total no. of cases	No. of cases fully vaccinated with IPD	Proportion of total cases evading the vaccine (%)
14(13v)	18	2	11.1
19A (13 v)	79	6	7.6
19F (13 v)	110	13	11.8
22F (23 v)	112	1	0.9
23F (13 v)	2	1	50.0
3(13v)	205	11	5.4

### Serotypes of public health concern

Due to the highly diverse nature of the population, separate phylogenetic trees were constructed for six sub-populations of public health concern to gain a better understanding or their individual population dynamics and identify any characteristics that make them more successful pathogens. These sub-populations of public health concern were identified based on their frequency, serotype inclusivity in vaccines and antibiotic susceptibility profile.

**Fig. 5.**
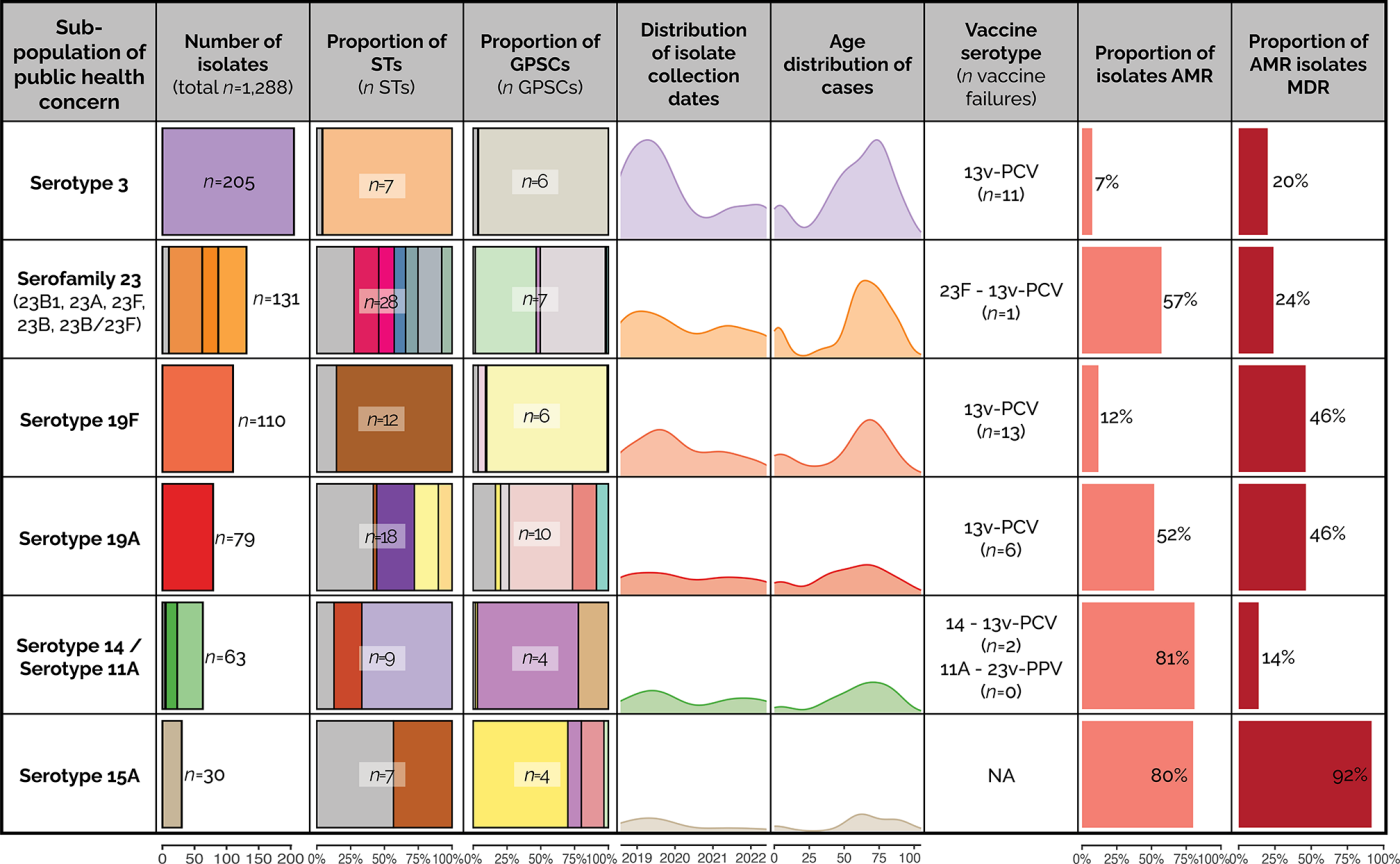
Key attributes of the sub-populations of public health concern. Sub-populations have been ordered based on the frequency of isolates. Scales for each of the data columns are displayed at the bottom of the figure. The number of isolates' column are coloured based on serotype, the proportion of ST is coloured based on ST and the proportion of global pneumococcal sequences cluster (GPSC) is coloured based on GPSC. Grey indicated minor types/clusters for all typing/clustering mechanisms. Serotypes from sub-populations of interest are listed if they contained in the 13-valent pneumococcal conjugate vaccine (PCV). The proportion of isolates that are classified as resistant to at least one of the antimicrobials (AMR) tested is listed, along with the proportion of total isolates that were resistant to three or more antimicrobial classes (MDR). Breakpoints are based on the 2022 CLSI guidelines. For detailed phylogenetic trees and associated metadata see Supplementary Material.

No sub-populations of public health concern were clustered in monophyletic clades associated with year of collection, suggesting they were not outbreak clones. For vaccine serotypes that were analysed in depth (3, 19F, 19A and 14), no phylogenetic association with vaccine failure events based on whole-genome alignments was observed. Though serotype 3 and 19F vaccine-failure events were mostly associated with one ST/GPSC (ST180/GPSC12 82 % vaccine failure events, *n*=9 and ST654/GPSC119 85 % vaccine failure events, *n*=11, respectively), they were also the most common ST/GPSC for these serotypes. All sub-populations of public health concern had similar case age distributions with a small peak at approximately 5 years of age and a larger peak at approximately 70 years (Figs 5 and S4–S9).

#### Serotype 3

Serotype 3 was investigated further as it was the most prevalent serotype, is contained in the 13v-PCV and had the most vaccine-failure events (*n*=11). Despite being a serotype included in the vaccine, there had been a steady increase in serotype 3 IPD cases until the reduction in all IPD cases in 2020 (Fig. S1a). Serotype 3 had very low levels of AMR (7 % resistant to any antibiotic) with only four of the isolates being classified as MDR. The level of genomic diversity was low for serotype 3 with the majority (95.6 %, *n*=196) being ST180 and GPSC12. The phylogeny of the ST180 isolates is split into four distinct clades of closely related isolates with one clade containing approximately 50 % of isolates (Figs 5 and S4).

#### Serofamily 23 (serotype 23A, 23F 23B and subtype 23B1)

Isolates in the serofamily 23 (serotype 23A, 23F 23B and subtype 23B1) were selected for further investigation as they had high rates of AMR/MDR (23A and subtype 23B1) and included STs with a high proportion of resistant isolates. No serofamily 23 serotypes are contained in the 13v-PCV. The number of IPD cases due to serofamily 23 had been increasing since 2012, declined in 2020 then started rising again (Fig. S1b). Serofamily 23 was very diverse at the ST level, containing 28 unique STs. However, the diversity at the GPSC level was much less with only seven clusters defined. All isolates within GPSC5 were resistant to penicillin (meningitis breakpoints), which included isolates from serotype 23A and subtype 23B1 and isolates from 11 STs. In contrast to subtype 23B1/GPSC5, serotype 23A/GPSC5 isolates were also resistant to macrolides. The majority of the remaining serofamily 23 (remaining 23A and 23B) isolates were GPSC7 and were mostly pan-susceptible (94.9 %, *n*=56) (Figs 5 and S5).

#### Serotype 19F

Serotype 19F was investigated further as the number of isolates per year continued to increase despite being contained in the 13v-PCV (Fig. S1c). Most isolates were ST654/GPSC119 (85.5 %, *n*=94) and pan-susceptible (four isolates resistant to at least one antibiotic). This serotype had the largest number of vaccine-failure events (*n*=13) and 12 occurred in GPSC119, of which 11 were ST654. The next most common ST in this serotype was ST179 (*n*=5) of which all isolates were resistant to penicillin (meningitis breakpoints) and three were MDR (Figs 5 and S6).

#### Serotype 19A

Serotype 19A was investigated further as it was a prevalent serotype and had a high proportion of AMR (isolates resistant to at least one of the antibiotics tested) and MDR isolates (52 and 44 %, respectively). Serotype 19A is contained in the 13v-PCV vaccine and following the vaccine’s introduction in 2011, the number of 19 A cases declined each year (Fig. S1d). Serotype 19A was one of the most diverse serotypes, containing 18 STs (equal with serotype 6C) and 10 GPSCs. All STs clustered together within the tree and there was a strong association between ST and AMR phenotype. Vaccine failure events (*n*=6) occurred in five unique STs. In contrast to the other sub-populations of public health concern, serotype 19A did not experience a marked decrease in cases in 2020 and 2021, case numbers remained consistent across the whole study period (Figs 5 and S7).

#### Serotype 11A/serotype 14

All isolates in serotype 11A and 14 were investigated further as both serotypes are contained in a pneumococcal vaccine (23v-PPV and 13v-PCV respectively), have a high proportion of AMR and 100 % of ST156 (any serotype) were resistant to penicillin (meningitis breakpoints). These two serotypes were investigated together as a large proportion of both serotypes were the same ST (ST156). All other non-serotype 11A/14 ST156 isolates were also included in the analysis to provide context. The number of IPD cases caused by serotype 14 and 11A remained consistent since 2011 and before decreasing in 2020 (Fig. S1e). Although serotype 14/ST156 isolates appeared to have broad-spectrum resistance to beta-lactams, serotype 11A/ST156 isolates were predominantly only resistant to penicillin. The majority of serotype 11A/GPSC6 (62.5 %, *n*=25) clustered more closely related to serotype 14 isolates on the phylogenetic tree than the remaining serotype 11A isolates. Serotype 14 had only two vaccine-failure events, both of which occurred in isolates of the same ST (ST156). These isolates were also resistant to a broad range of beta-lactams (Figs 5 and S8).

#### Serotype 15A

The number of IPD cases caused by serotype 15A remained consistent since 2012 and before decreasing in 2020 (Fig. S1e). Serotype 15A was investigated further as it had a high proportion of AMR (80 %) and all AMR isolates were MDR isolates. Despite this, the majority of MDR isolates were resistant to penicillin only and susceptible to all other beta-lactams. ST1591 (*n*=4) isolates were resistant to many antibiotics, with MICs in the resistant or intermediate classification for all beta-lactam antibiotics amoxicillin (Figs 5 and S9).

## Discussion

The use of whole-genome sequencing technology is rapidly being adopted globally for public health surveillance of pathogens due to the high resolution and unparalleled detail it can provide. However, to embed genomics-enabled public health surveillance on a routine basis, a framework needs to be established for each pathogen. Limited routine genomic surveillance studies have been performed on *Streptococcus pneumoniae* genomics in Australia, with previous analysis focusing only on a few key serotypes [36]. Here, we performed an in-depth genomic analysis of all IPD isolates received by the state public health laboratory over a 4 year period. The proportion of IPD cases formally notified to the National Notifiable Disease Surveillance System (NNDSS) that had sequence data available was high (~80 %) and hence the sampling can be considered representative of the *S. pneumoniae* IPD causing population. We identified six sub-populations of public health concern (based on high prevalence despite serotype inclusion in current vaccines, along with high rates of AMR) and described the heterogeneity in the genomic structure of each sub-population.

Despite the 13vPCV vaccine being introduced nearly 10 years ago, three of the most prevalent serotypes in this study were vaccine serotypes (3, 19A and 19F). The prevalent non-vaccine serotypes identified in our study (serotypes 22F, 9 N and 6C) are consistent with other studies [37, 38]. However, a recent study of international pneumococcal genomics by Lo *et al.* showed the five most prevalent serotypes in the 13vPCV period varied between countries with only six serotypes common to two or more countries [38]. In addition, the same prevalent non-vaccine serotypes could be associated with distinctive lineages and dissimilar antibiotic resistance profiles in different countries [38]. This necessitates each country to monitor the local *S. pneumoniae* population and cannot simply draw conclusion from nearby jurisdictions.

Despite occurring most frequently, serotype 3 had less genomic diversity when compared to other serotypes, as well as less antibiotic resistance, consistent with a largely clonal population. The majority of serotype 3 isolates were ST180 and GPSC5, consistent with other studies [39, 40]. Its continued success, despite high levels of vaccination coverage, is likely due to the low efficacy of the vaccine rather than a heterogenous genome or *cps* locus. The low efficacy of the PCV against serotype 3 is well documented, with the lack of protection suggested to be related to the synthase-dependent synthesis of the capsular polysaccharide, resulting in polysaccharide that is not linked to peptidoglycan and thus reducing the efficacy of the antibody-mediated immune response [41].

In contrast, the heterogeneity of serotype 19 (19A and 19F), considering both the ST and *cps* locus, are thought to contribute to the high vaccine-failure rate [36, 42]. Although the ST and GPSC diversity of serotype 19A isolates in this study supported this, serotype 19F was comparatively clonal. It is hypothesized that the vaccine-failure cases of 19F could instead be due to heterogeneity of the *cps* locus, something that was not measured in this study.

Of particular concern in serofamily 23 was subtype 23B1. This serotype can only be identified using WGS as there is no distinction between serotype 23B and 23B1 serologically or in terms of polysaccharide structure [43]. All subtype 23B1 isolates (*n*=52) were resistant to penicillin (oral breakpoints) while only one serotype 23B (*n*=23) was resistant. All subtype 23B1 isolates were GPSC5, a lineage that has been well documented internationally to have high rates of penicillin resistance and has also been associated with serofamily 23 (mainly serotype 23A) [20, 37]. The increased resolution of whole-genome-based pneumococcal serotyping compared to Quellung enables enhanced distinction of serotypes, some of which are important as they are associated with higher levels of antimicrobial resistance. This reinforces the benefits of using WGS for enhanced surveillance of IPD. We present our data validating seroBA for *in silico* serotyping to facilitate ISO-accreditation of this test in routine use in a public health reference laboratory, another important step in the implementation of genomics for public health surveillance.

Despite having the highest rate of AMR and MDR of any sub-population of public concern investigated, all 15A isolates collected over the study period were susceptible to penicillin using oral breakpoints, suggesting that first-line treatments would still be effective against this serotype. Our results are in contrast to a recent study by Hawkins *et al.*, which showed that the serotype 15A/clonal complex 63 (CC63) is mostly penicillin non-susceptible [44]. The study also describes large genomic overlaps between the serotype 14 and 15A populations and suggests that 15 A/CC63 emerged from a serotype 14 ancestor prior to the development of pneumococcal vaccines. Though our isolates are all collected at least 10 years after the introduction of the 7vPCV, we observed no overlap between the serotype 14 and 15A populations. The serotype 15A isolates we describe still present a large public health risk, as a combined serotype and penicillin-binding protein (*pbp*) switching event could lead to an extensively drug-resistant virulent serotype isolate. The shared ST of serotype 19A/ST63 (*n*=2) and 15A/ST63 (*n*=13) isolates, in addition to their similar AMR profile, is suggestive of a serotype switching event. Multiple examples of vaccine evasion via serotype switching to 15A (a serotype not contained in the either the 13v-PCV or the 23v-PPV) have been documented [45, 46]. Although serotype 15B is contained in the 23v-PPV and is to be included in other PCVs in development that contain a broader selection of serotypes, 15B immunity has not been shown to provide cross-protection to 15A [47].

To effectively monitor changing trends in IPD genomics, it is essential to be able to provide global context. The Global Pneumococcal Sequencing Clusters (GPSCs), curated by the Global Pneumococcal Sequencing Project, provide an easy and effective way of doing this. The GPSC6 lineage contains multiple STs (predominantly ST156) and serotypes (both 13v-PCV and non-13v-PCV but predominantly serotype 14 and 11A) but is united by a penicillin-resistant phenotype. This lineage has been observed in multiple other countries, with international studies commenting on its ability to adapt and continue to cause infection under vaccine and antimicrobial pressure [20, 48]. Despite the utility of the GPSC scheme, we could not identify any Australian pneumococcal sequences in the databases used to generate the typing scheme [20]. This therefore raises questions about the applicability of the scheme and its ability to distinguish genomic backgrounds unique to Australia. As WGS continues to become more routine in Australia, more specifically Victoria, it is imperative that data is made available to public databases to ensure that the scheme includes representative sequences from all regions, where available, to ensure global representativeness and generalizability.

In addition, we have identified many discrepancies between AMR genotype and phenotype when using AbritAMR and the AMRFinderPlus Reference Gene Catalogue, particularly for beta-lactam antibiotics. This is due to the method the tool uses not being able to identify novel resistant alleles of the penicillin-binding proteins (pbps), the genes conferring resistance to beta-lactam antibiotics. Rather, the tool can only identify if an isolate has an allele that is not a known susceptible allele. Alternative methods that are able to categorize isolates with novel alleles and take into account the accumulative nature of the AMR determinants should be investigated further, such as those developed by Demczuk *et al.* [49].

In conclusion, we used whole-genome sequencing to provide a comprehensive description of *S. pneumoniae* isolated from Victorian IPD cases. We highlight the value of genomics is resolving population dynamics within a serotype and the large heterogeneity between serotypes, which may have implications for future vaccine development. Within the highly diverse pneumococcal population, we identified six sub-populations of public health concern (serotypes 3, 19F, 19A, 14, 11A, 15A and serofamily 23) based on high prevalence despite serotype inclusion in current vaccines, along with high rates of AMR. Our study provides a genomics framework for the ongoing surveillance of IPD and identifies sub-populations of public health concern that can form that focus of continued genomic surveillance.

## Supplementary Data

Supplementary material 1

Supplementary material 2

Supplementary material 3

Supplementary material 4
