# Supplementary material for: Population structure, serotype distribution and antibiotic resistance of Streptococcus pneumoniae causing invasive disease in Victoria, Australia
Source: Microb Genom. 2023 Jul 20;9(7):mgen001070. doi: 10.1099/mgen.0.001070 (PMC10438814; doi:10.1099/mgen.0.001070)
Supplement: Supplementary material 1 [file mgen-9-1070-s001.pdf]

Supplementary Materials for

Population structure, serotype distribution and antibiotic resistance of Streptococcus pneumoniae causing invasive disease in Victoria, Australia.

Charlie Higgs, Lamali Sadeesh Kumar, Kerrie Stevens, Janet Strachan, Norelle L. Sherry, Kristy Horan, Josh Zhang, Timothy P. Stinear, Benjamin P. Howden, Claire L. Gorrie.

The Supplementary Materials include:

Supplementary Table 1 – 3
Supplementary Figures 1 – 9
Legends for Supplementary Data 1 – 3

Other Supplementary Materials for this manuscript include the following:

Supplementary Data 1 – 3

Supplementary Figures

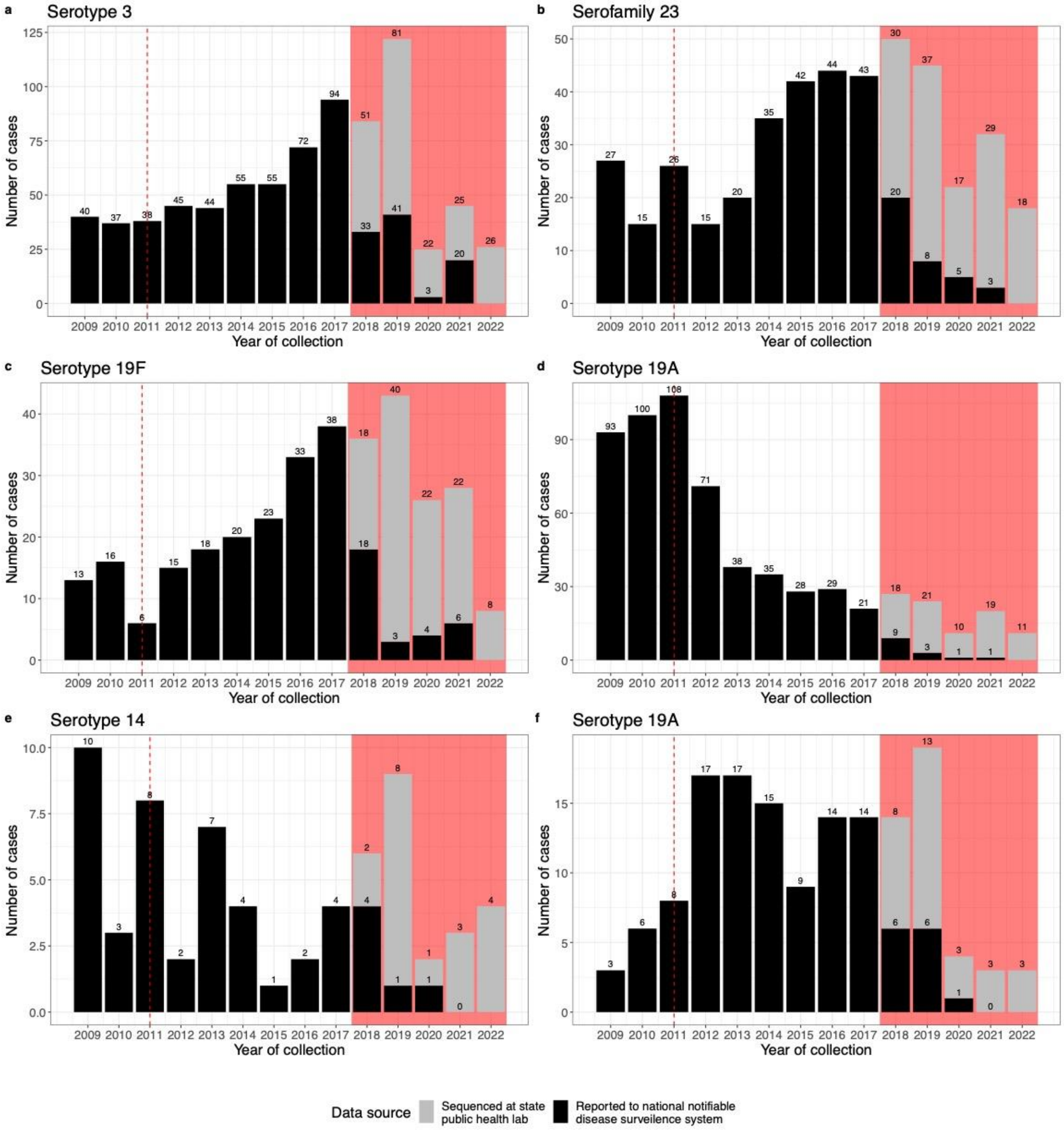

Supplementary Figure 1: Count of invasive pneumococcal disease (IPD) cases over time separated by sub populations of public health concern. The sub populations of public health concern include a) serotype 3, b) serofamily 23, c) serotype 19F, d) serotype 19A, e) serotype 14 and 11A and f) serotype 15A. Case counts are based on IPD cases reported to the National Notifiable Disease Surveillance System (NNDSS) by the Victorian Department of Health and the number of Streptococcus pneumoniae isolates sequenced at the state public health included as part of this study (subset of the NNDSS number). Note that for NNDSS data the state totals for Victoria and Tasmania are grouped together and the notification data is not available for 2022. Shaded red area indicates the study period (July 2018 to Jun 2022). Dashed red line indicated when the Australian vaccine schedule was updated to include the 13-valent pneumococcal conjugate vaccine in 2011 (previously the 7-valent vaccine was used).

Type of result	Quellung Serotype	SeroBA Serotype	n
**Discordant results**	15C	15B	4
	15B	15C	3
	11A	11E	2
	38	25A	2
	11C	11A	1
	11F	11B	1
	12A	12B	1
	13	15B	1
	15C	19A	1
	18F	18B	1
	19A	15B	1
	19A	22F	1
	22F	19A	1
	23B	23F	1
	3	8	1
	33F	33A	1
	7B	7C	1
	7F	9L	1
	9A	9L	1
	**Total**	**Total**	**26**
**Concordant result**	**Total**	**Total**	**739**

Supplementary Table 1: Count of results between the Quellung Reaction serotyping and *in silico* serotyping using SeroBA. Note: the serotypes 23B and 6B have subtypes (23B1 and 6E) that can only be detected using in silico prediction as they are not serologically distinct from each other. There were isolates identified as 23B that were 23B1 (n=33) and 6B that were 6E (n=1) but these were not classed as discrepancies.

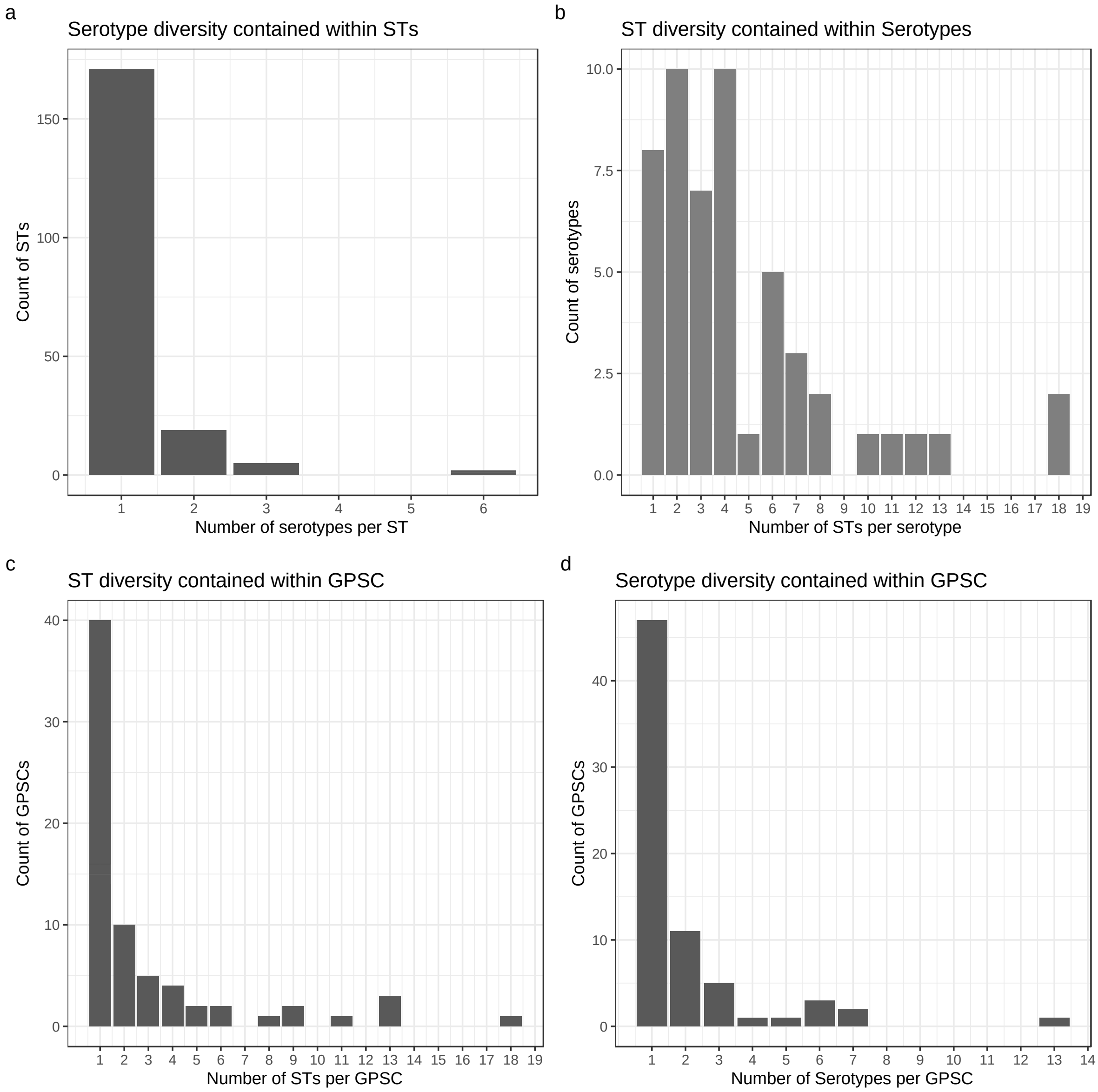

Supplementary Figure 2: Comparison between diversity within typing mechanisms for *Streptococcus pneumoniae*. a) Serotypes contained within sequence types (STs), b) STs contained within serotypes, c) STs diversity contained within global pneumococcal sequence clusters (GPSCs) and d) serotypes contained within GPSCs.

Serotype	Total isolates n	Number resistant isolates (≥1 antibiotic)	% Resistant isolates (≥1 antibiotic)	Number MDR isolates	% Resistant isolates that are MDR
**3**	205	15	7	3	20
**22F**	112	2	2	0	0
**19F**	110	13	12	6	46
**09N**	100	10	10	1	10
**19A**	79	41	52	19	46
**06C**	61	17	28	4	24
**23B1**	52	51	98	0	0
**33A**	49	29	59	0	0
**8**	47	3	6	0	0
**23A**	44	16	36	13	81
**12F**	41	13	32	4	31
**11A**	40	28	70	3	11
**15A**	30	24	80	22	92
**16F**	29	5	17	1	20
**15B**	27	5	19	1	20
**23B**	25	4	16	1	25
**10A**	22	7	32	0	0
**07F**	20	1	5	0	0
**35B**	19	9	47	0	0
**14**	18	18	100	3	17
**35F**	16	2	13	0	0
**31**	13	1	8	0	0
**17F**	12	2	17	0	0
**24B**	10	3	30	0	0

Supplementary Table 2: Number of isolates resistant to at least one antibiotic and multi drug resistant (MDR) in serotypes with ten or more isolates over the study period. MDR isolates were defined as resistant to three or more antimicrobial classes. Breakpoints are based on the 2022 CLSI guidelines.

ST	Total isolates	Number Resistant isolates (≥1 antibiotic)	% Resistant isolates
**180**	196	13	7
**654**	94	4	4
**66**	87	4	5
**433**	76	2	3
**156**	42	42	100
**53**	42	2	5
**717**	26	26	100
**439**	24	3	13
**1373**	23	23	100
**218**	21	1	5
**191**	18	1	6
**100**	17	1	6
**2068**	17	5	29
**1692**	15	1	7
**4237**	15	10	67
**63**	15	14	93
**2042**	14	1	7
**2062**	14	13	93
**62**	14	2	14
**162**	13	7	54
**1684**	12	1	8
**2372**	12	12	100
**338**	11	11	100
**1349**	10	10	100

Supplementary Table 3: Number of isolates resistant to at least one antibiotic in sequence types (STs) with ten or more isolates over the study period. Breakpoints are based on the 2022 CLSI guidelines.

**Antibiotic Subclass**	**Antibiotic** (Men/Non Men breakpoint used, if applicable)	**Total number isolates tested**	**Resistant phenotype N** (%)	**Major error N** (%)	**Very Major error N** (%)	**Determinants** (% of resistance explained)
Beta-Lactam	Amox-Clav Acid	1176	14 (1.2%)	270 (23%)	1 (7.1%)	pbp1a*;pbp2b* (14%), pbp1a*;pbp2b*;pbp2x* (36%), pbp2b*;pbp2x* (43%)
	Cefepime (Men)	1286	40 (3.1%)	230 (17.9%)	0 (0%)	pbp1a*;pbp2b* (8%), pbp1a*;pbp2b*;pbp2x* (78%), pbp2b*;pbp2x*
	Cefepime (Non Men)	1286	4 (0.3%)	270 (21%)	0 (0%)	pbp1a*;pbp2b*;pbp2x* (100%)
	Cefotaxime (Men)	1286	24 (1.9%)	243 (18.9%)	0 (0%)	pbp1a*;pbp2b* (8%), pbp1a*;pbp2b*;pbp2x* (71%), pbp2b*;pbp2x* (21%)
	Cefotaxime (Non Men)	1286	0 (0%)	286 (22.2%)	0 (0%)	
	Ceftriaxone (Men)	1285	39 (3%)	228 (17.7%)	1 (2.6%)	pbp1a*;pbp2b* (5%), pbp1a*;pbp2b*;pbp2x* (77%), pbp2b*;pbp2x* (15%)
	Ceftriaxone (Non Men)	1285	4 (0.3%)	272 (21.2%)	0 (0%)	pbp1a*;pbp2b*;pbp2x* (100%)
	Cefuroxime	1286	77 (6%)	216 (16.8%)	0 (0%)	pbp1a*;pbp2b*
	Ertapenem	1286	0 (0%)	302 (23.5%)	0 (0%)	
	Meropenem	1286	22 (1.7%)	245 (19.1%)	0 (0%)	pbp1a*;pbp2b* (5%), pbp1a*;pbp2b*;pbp2x* (64%),
	Penicillin (Men)	1285	261 (20.3%)	65 (5.1%)	16 (6.1%)	pbp1a* (<1%),
	Penicillin (Non Men)	1285	4 (0.3%)	276 (21.5%)	0 (0%)	pbp1a*;pbp2b*;pbp2x* (50%),
	Penicillin (Oral)	1285	65 (5.1%)	65 (5.1%)	0 (0%)	pbp1a*;pbp2b*
Chloramphenicol	Chloramphenicol	1286	21 (1.6%)	0 (0%)	9 (42.9%)	cat-TC*^ (5%), catA (52%)
Macrolide	Azithromycin	1135	123 (10.8%)	3 (0.3%)	27 (22%)	erm(B) (72%),
	Clindamycin	1286	106 (8.2%)	9 (0.7%)	7 (6.6%)	erm(B) (88%),
	Erythromycin	1286	145 (11.3%)	3 (0.2%)	36 (24.8%)	erm(B) (70%), erm(B)* (4%), erm(B)*^ (1%)
Quinolone	Levofloxacin	1285	2 (0.2%)	1280 (99.6%)	0 (0%)	parC_S79F;pmrA* (50%),
	Moxifloxacin	1285	1 (0.1%)	1279 (99.5%)	0 (0%)	pmrA (100%)
Tetracycline	Tetracycline	1286	135 (10.5%)	19 (1.5%)	11 (8.1%)	tet(32) (6%), tet(M) (39%), tet(M)* (47%)

Supplementary Table 4: Comparison of AMR phenotype and genotype. Major error = number (%) of susceptible isolates in which a resistance determinant was identified; very major error = number (%) of resistant isolates in which a resistance determinant was not identified (i.e., unexplained resistance). Genes recovered with >50% but <90% coverage of a gene in the gene catalog will be annotated with ^. Genes annotated with * indicate >90% coverage and > identity threshold < 100% identity.

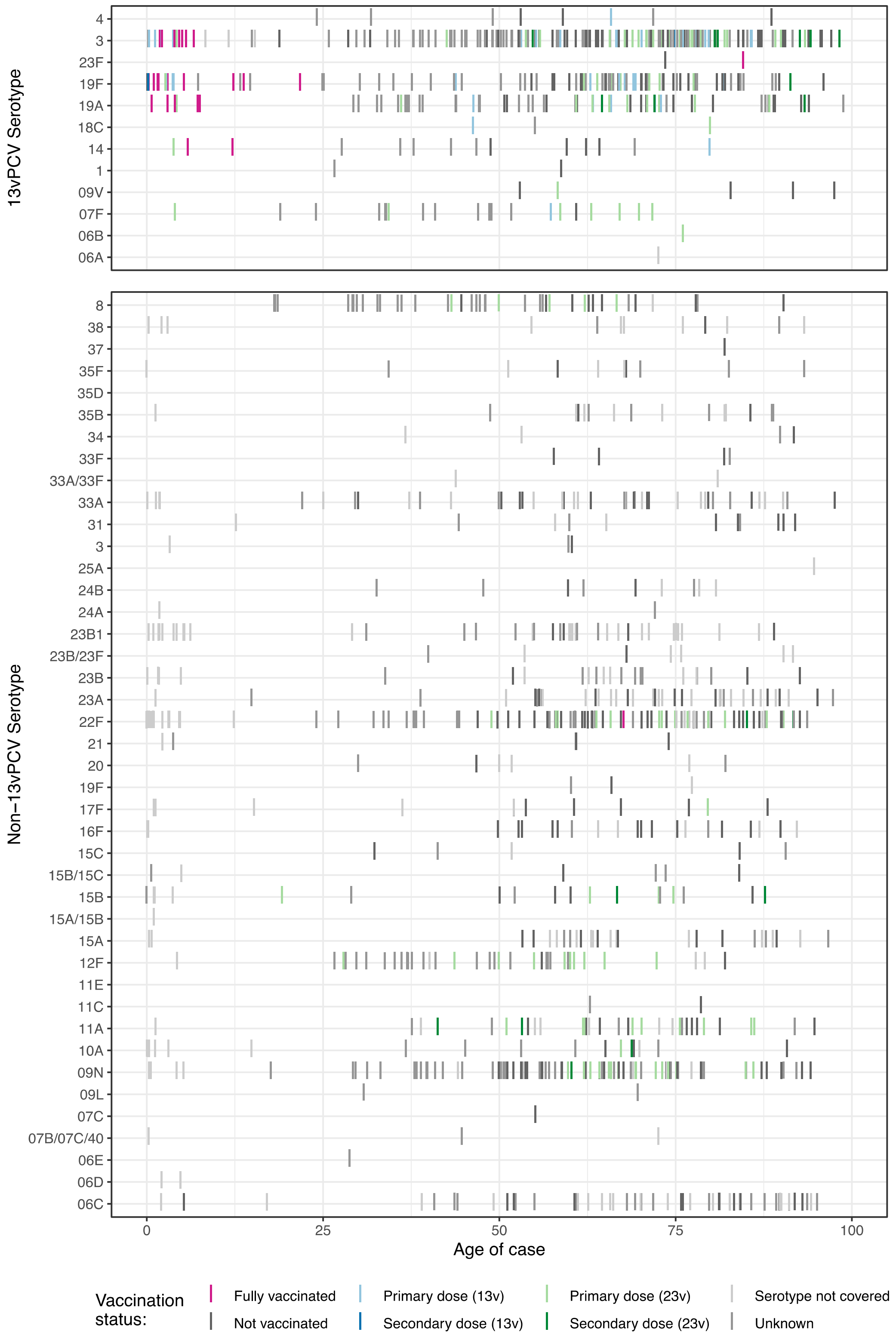

Supplementary Figure 3: Age distribution of IPD cases by serotype. Vaccination status was only available for a subset of isolates (June 2018 and December 2021, n=1,118). Vaccination type is either the 13-valent pneumococcal conjugate vaccine (13v) or the 23-valent pneumococcal polysaccharide vaccine (23v).

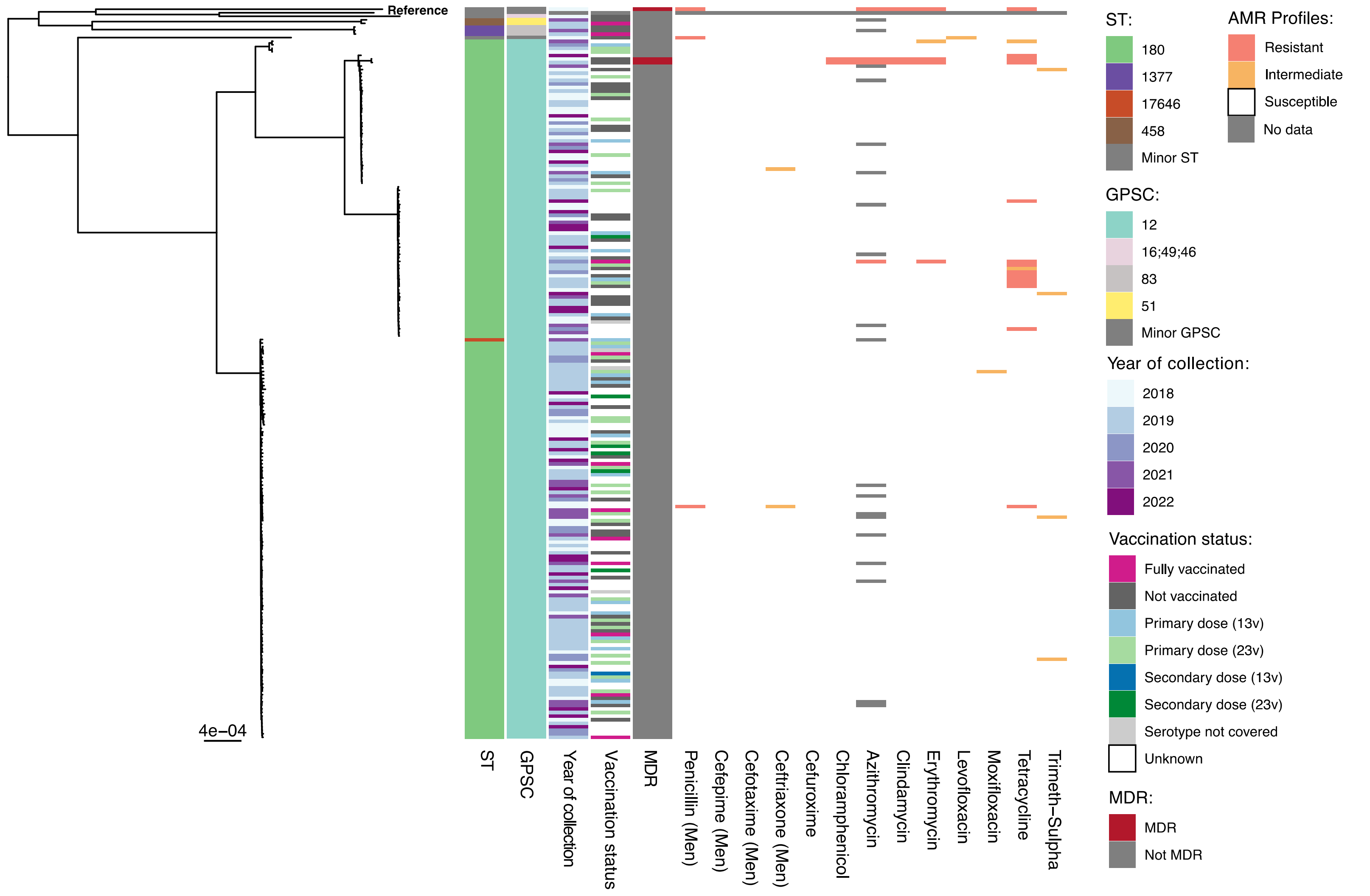

Supplementary Figure 4: Midpoint-rooted maximum-likelihood phylogenetic tree of all serotype 3 isolates (n=205). Minor sequence types (STs) and global pneumococcal sequence cluster (GPSC) were defined as those that contained less than 10 isolates over the study period. Vaccination status was obtained for a subset of isolates (collected between July 2018 and December 2021). Vaccination type is either the 13-valent pneumococcal conjugate vaccine (13v) or the 23-valent pneumococcal polysaccharide vaccine (23v). Antimicrobial susceptibility testing breakpoints are based on the 2022 CLSI guidelines. Multidrug resistance (MDR) was defined as being resistant to three or more classes of antibiotics. The reference was ASM966447v1 (GCA_009664475.1); collected 2014, serotype 19A, ST199.

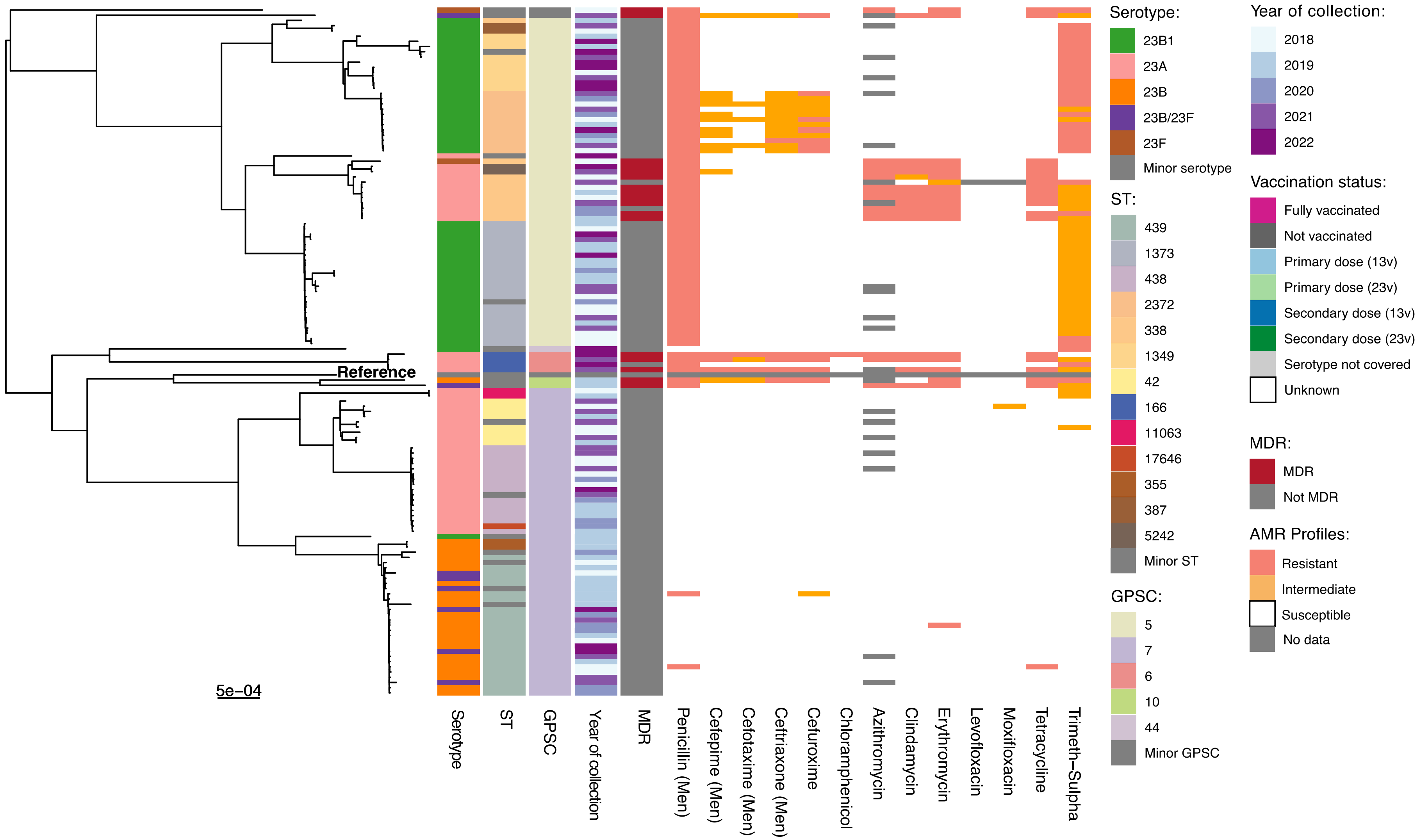

Supplementary Figure 5: Midpoint-rooted maximum-likelihood phylogenetic tree of all serofamily 23 isolates (serotype 23B1, 23A, 23F, 23B, 23B/23F, n=131). Minor sequence types (STs) and global pneumococcal sequence cluster (GPSC) were defined as those that contained less than 10 isolates over the study period. Vaccination status was obtained for a subset of isolates (collected between July 2018 and December 2021). Vaccination type is either the 13-valent pneumococcal conjugate vaccine (13v) or the 23-valent pneumococcal polysaccharide vaccine (23v). Antimicrobial susceptibility testing breakpoints are based on the 2022 CLSI guidelines. Multidrug resistance (MDR) was defined as being resistant to three or more classes of antibiotics. The reference was ASM966447v1 (GCA_009664475.1); collected 2014, serotype 19A, ST199.

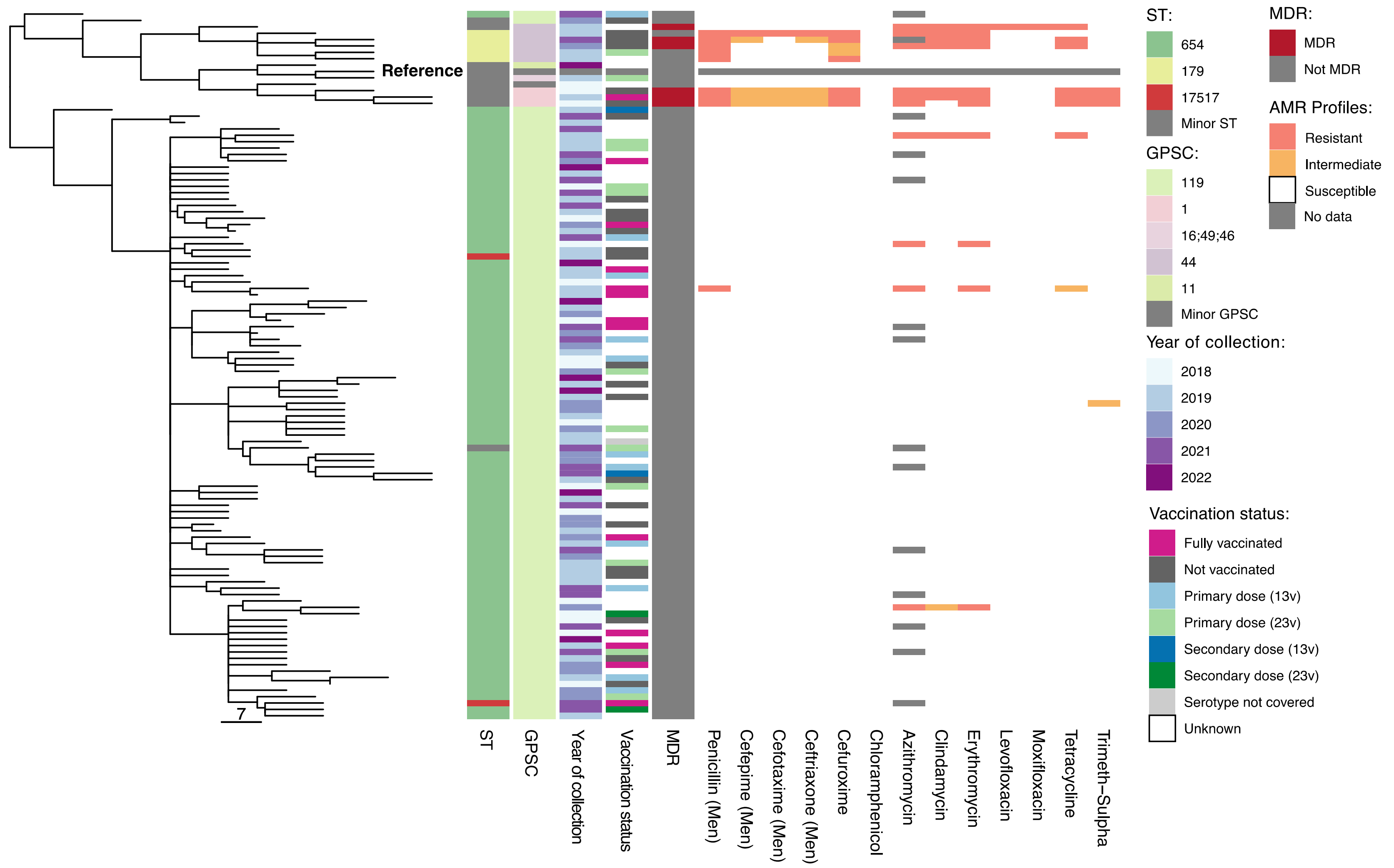

Supplementary Figure 6: Midpoint-rooted maximum-likelihood phylogenetic tree of all serotype 19F isolates (n=110). Minor sequence types (STs) and global pneumococcal sequence cluster (GPSC) were defined as those that contained less than 10 isolates over the study period. Vaccination status was obtained for a subset of isolates (collected between July 2018 and December 2021). Vaccination type is either the 13-valent pneumococcal conjugate vaccine (13v) or the 23-valent pneumococcal polysaccharide vaccine (23v). Antimicrobial susceptibility testing breakpoints are based on the 2022 CLSI guidelines. Multidrug resistance (MDR) was defined as being resistant to three or more classes of antibiotics. The reference was ASM966447v1 (GCA_009664475.1); collected 2014, serotype 19A, ST199.

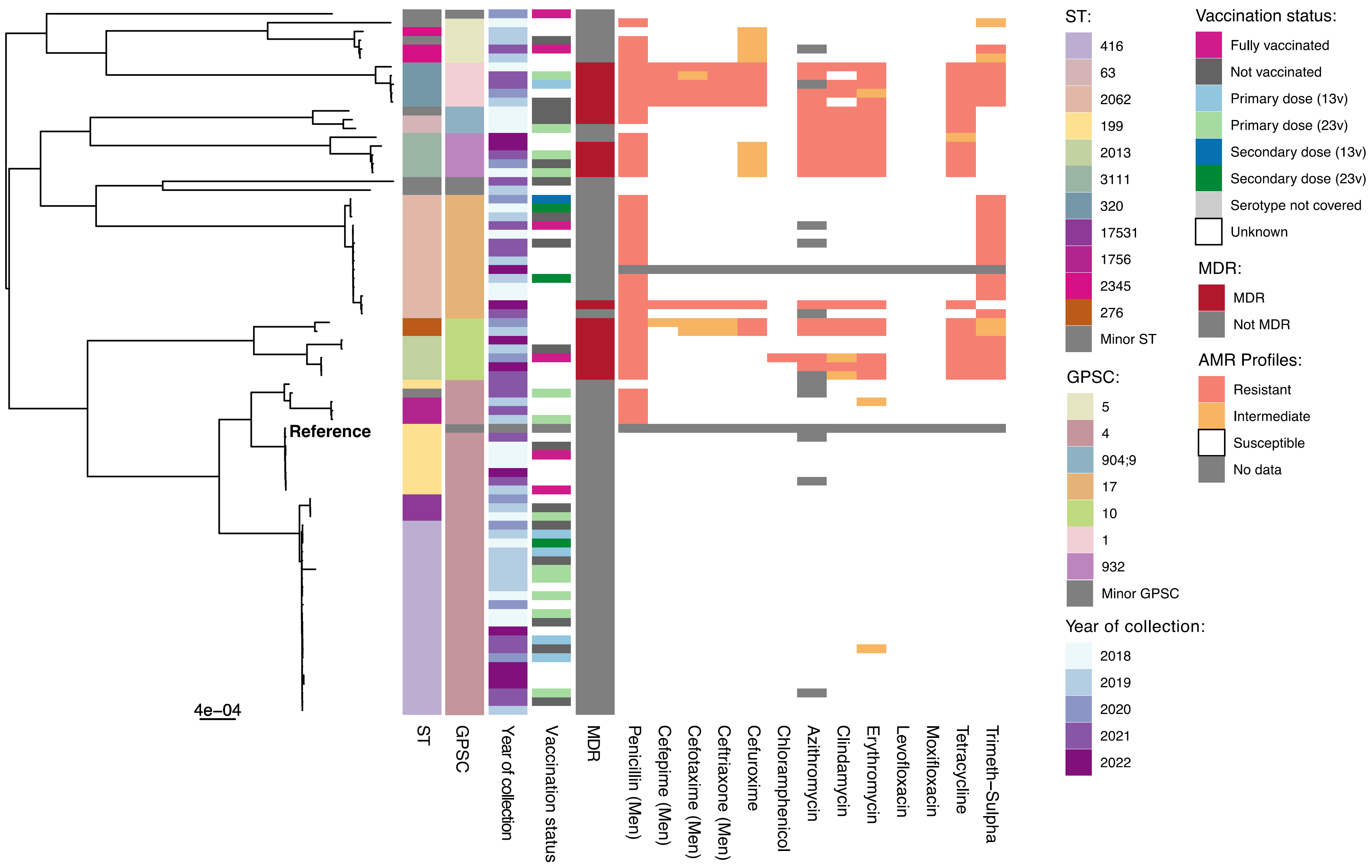

Supplementary Figure 7: Midpoint-rooted maximum-likelihood phylogenetic tree of all serotype 19A isolates (n=79). Minor sequence types (STs) and global pneumococcal sequence cluster (GPSC) were defined as those that contained less than 10 isolates over the study period. Vaccination status was obtained for a subset of isolates (collected between July 2018 and December 2021). Vaccination type is either the 13-valent pneumococcal conjugate vaccine (13v) or the 23-valent pneumococcal polysaccharide vaccine (23v). Antimicrobial susceptibility testing breakpoints are based on the 2022 CLSI guidelines. Multidrug resistance (MDR) was defined as being resistant to three or more classes of antibiotics. The reference was ASM966447v1 (GCA_009664475.1); collected 2014, serotype 19A, ST199.

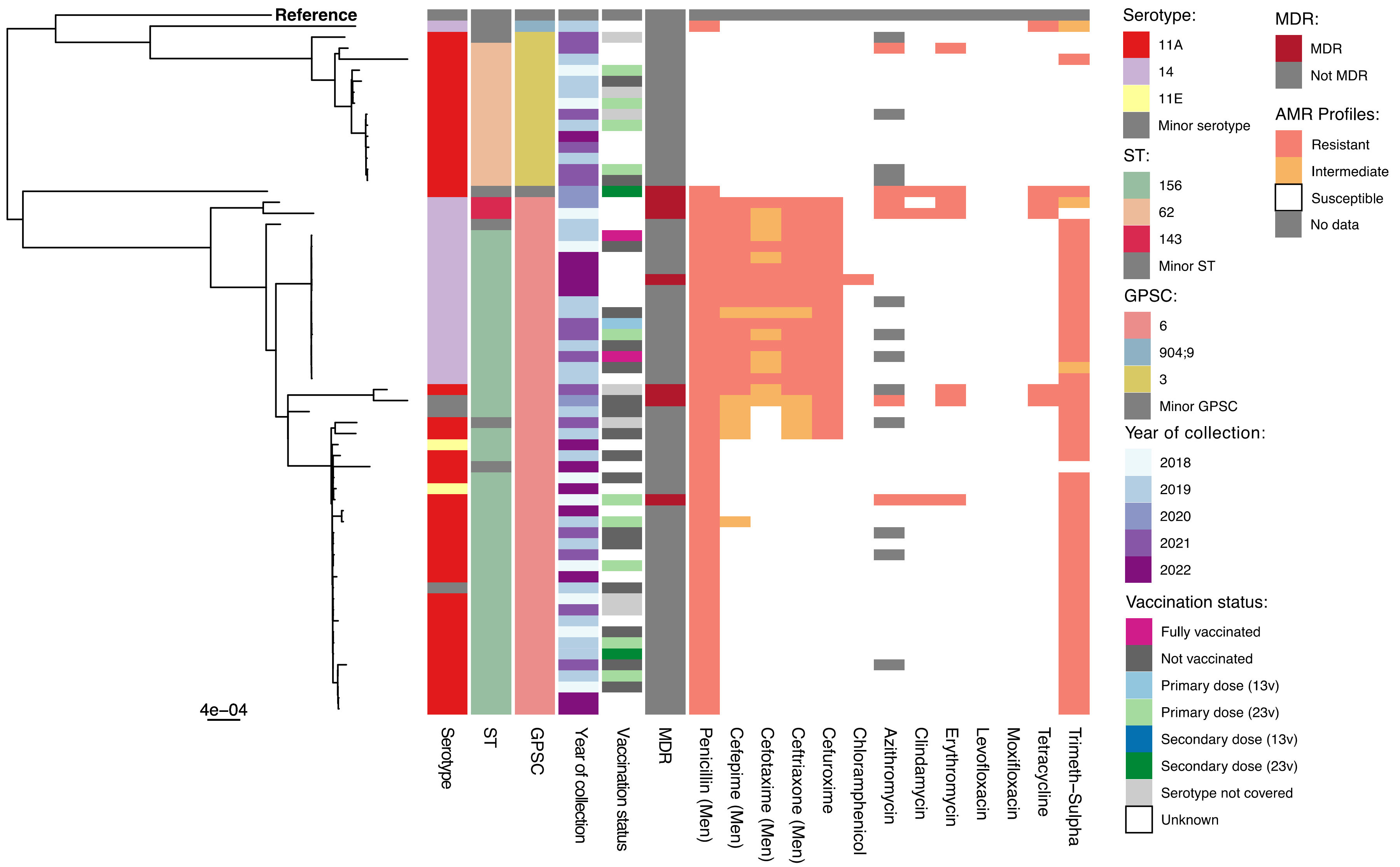

Supplementary Figure 8: Midpoint-rooted maximum-likelihood phylogenetic tree of all serotype 14/serotype 11A/ST156 isolates (n=63). Minor sequence types (STs) and global pneumococcal sequence cluster (GPSC) were defined as those that contained less than 10 isolates over the study period. Vaccination status was obtained for a subset of isolates (collected between July 2018 and December 2021). Vaccination type is either the 13-valent pneumococcal conjugate vaccine (13v) or the 23-valent pneumococcal polysaccharide vaccine (23v). Antimicrobial susceptibility testing breakpoints are based on the 2022 CLSI guidelines. Multidrug resistance (MDR) was defined as being resistant to three or more classes of antibiotics. The reference was ASM966447v1 (GCA_009664475.1); collected 2014, serotype 19A, ST199.

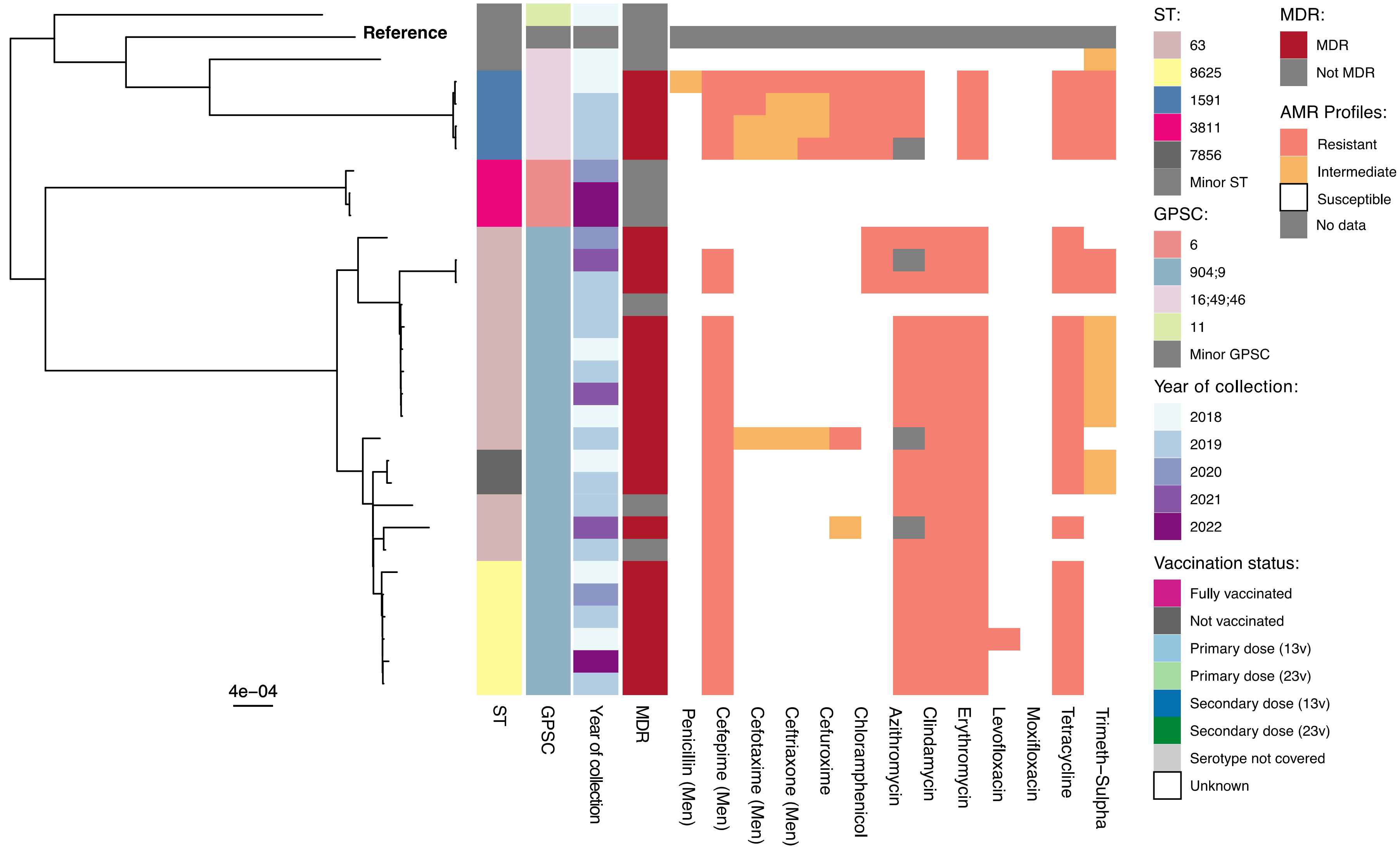

Supplementary Figure 9: Midpoint-rooted maximum-likelihood phylogenetic tree of all serotype 15A isolates (n=30). Minor sequence types (STs) and global pneumococcal sequence cluster (GPSC) were defined as those that contained less than 10 isolates over the study period. Vaccination status was obtained for a subset of isolates (collected between July 2018 and December 2021). Vaccination type is either the 13-valent pneumococcal conjugate vaccine (13v) or the 23-valent pneumococcal polysaccharide vaccine (23v). Antimicrobial susceptibility testing breakpoints are based on the 2022 CLSI guidelines. Multidrug resistance (MDR) was defined as being resistant to three or more classes of antibiotics. The reference was ASM966447v1 (GCA_009664475.1); collected 2014, serotype 19A, ST199.

Supplementary data:

Supplementary Data 1: Isolate list and metadata for all isolates, including AST results.

Supplementary Data 2: Count of IPD cases over time by serotype national notifiable disease surveillance system (NNDSS).

Supplementary Data 3: Results from the validation of the *in silico* serotyping method.
